# HDACs targeting from traditional Chinese medicine: mechanisms and therapeutic potential in rheumatoid arthritis

**DOI:** 10.3389/fphar.2026.1765295

**Published:** 2026-03-19

**Authors:** Caiwen Sun, Shaowa Lv

**Affiliations:** Key Laboratory of Basic and Application Research of Beiyao, Heilongjiang University of Chinese Medicine, Harbin, China

**Keywords:** epigenetics, histone deacetylase inhibitor, histone deacetylase activator, rheumatoid arthritis, traditional chinese medicine

## Abstract

Rheumatoid arthritis (RA) is a complex autoimmune disease driven by dysregulated epigenetic mechanisms, with histone deacetylases (HDACs) playing isoform-specific pathogenic or protective roles by modulating histone acetylation, immune function, and bone metabolism. While HDAC modulators (inhibitors/activators) show therapeutic potential for RA, current single-target agents are limited by poor isoform selectivity, off-target toxicity, and inadequate attenuation of RA’s multi-pathway pathology. In addition, the frequent occurrence of pan-assay interference compounds in natural-product space necessitates rigorous validation to reduce false-positive bioactivity claims. In this narrative review, we focus on Traditional Chinese Medicine (TCM)-derived metabolites as promising HDAC modulators, systematically summarizing recent progress in their development for RA. We highlight TCM-derived inhibitors, activators, and dual modulators, along with their isoform-specific mechanisms and structural basis of HDAC interaction. Notably, TCM-derived modulators exert synergistic effects by targeting multiple pathological links in RA, including synovial inflammation, fibroblast-like synoviocyte proliferation, immune imbalance, and bone destruction. However, their clinical translation is hindered by insufficient human clinical data, inconsistent TCM standardization, and limited pharmacokinetic/pharmacodynamic characterization. This review bridges traditional botanical medicine with modern epigenetic drug development, providing insights into RA therapies. Future research should prioritize clinical validation, standardized formulation, and mechanistic refinement to advance TCM-derived HDAC modulators toward clinical application.

## Graphical Abstract

**Figure:**
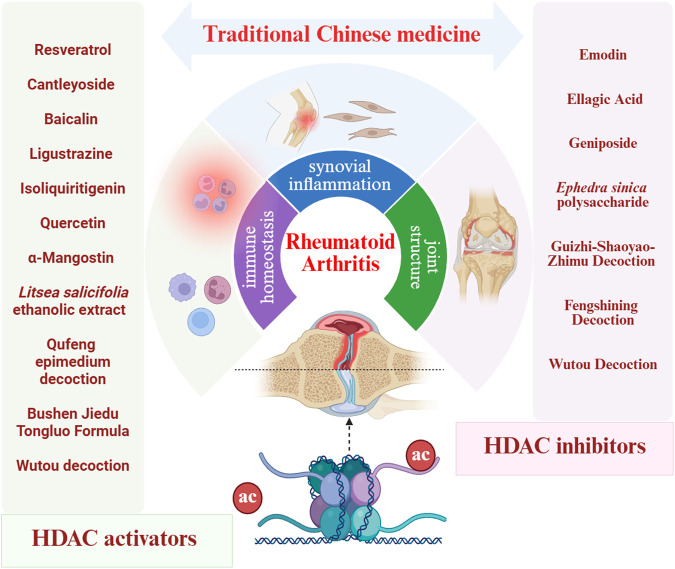

## 1 Introduction

RA, a persistent inflammatory condition of autoimmune origin marked by synovial joint swelling, has a global incidence of 0.5%–1% and is one of the major refractory diseases requiring urgent solutions in the global medical community (Zhang et al., 2024). The pathological progression of RA is highly complex, with synovitis as its initial and most critical pathological feature (Abuwarwar et al., 2018). During synovitis progression, synovial tissue is extensively infiltrated by inflammatory cells. At the same time, synovial fibroblasts undergo abnormal proliferation, leading to significant hyperplasia of the synovial lining layer. Proliferative synovial tissue secretes large amounts of pro-inflammatory mediators, which drive the progressive degradation of joint cartilage and bone tissue. This ultimately impairs joint structure and function, causing severe pain and substantial discomfort to patients (Neofotistou-Themeli et al., 2024). To date, despite extensive and in-depth research on RA among medical researchers, its exact cause still lacks consensus. Given the complexity of causes and the diversity of its clinical symptoms, identifying direct therapeutic targets for RA has become a key step in developing novel drugs and breaking through the limitations of existing treatments.

Studies have confirmed that the expression of various HDACs in FLS from RA patients presents is markedly dysregulated (Karami et al., 2020). HDACs, as key epigenetic regulatory enzymes, regulate gene expression by reducing histone acetylation levels, thereby influencing chromatin structure and function (Hawtree et al., 2013). HDACis can effectively reverse this aberrant histone modification state, alter chromatin conformation, regulate chromatin-binding protein functions, and induce cell cycle arrest and apoptosis, thereby suppressing the abnormal proliferation and invasive behavior of FLS and inhibiting the pathological progression of RA. Recent studies have confirmed that HDACis exert significant anti-inflammatory effects in RA models, reducing joint inflammation and delaying bone destruction (Vijaykrishnaraj et al., 2023). In addition to inhibitors, emerging evidence suggests that selective HDAC activators could play a role in modulating inflammatory signaling pathways by regulating the enzymatic activity of specific HDAC isoforms, though their precise mechanisms in RA remain to be fully elucidated. Both HDACi and activators offer the advantage of targeting the core mechanisms underlying dysregulated gene expression, with inhibitors acting to restore aberrant acetylation patterns and activators fine-tuning enzyme activity to rebalance epigenetic homeostasis (Costanzo et al., 2025). These findings provide a robust theoretical foundation for targeting HDACs as a potential therapeutic strategy in RA.

Currently, drug development targeting HDACs still faces substantial challenges. While virtual screening can improve efficiency, it is constrained by the size and chemical diversity of metabolite libraries, and this limitation that makes it prone to generating false positives or false negatives. Single-target screening struggles to reflect the complex *in vivo* biological environment, whereas multi-target screening, despite offering advantages, significantly increases the complexity of experimental design and validation (Ogbonna et al., 2025). Furthermore, some HDAC isoforms share highly similar sequences, creating difficulties in selective targeting, which in turn impairs the target specificity and delivery efficiency of inhibitors. Collectively, these factors hinder the development of HDAC-targeted drugs. In this context, TCM offers a new research perspective for regulating HDAC-related pathways, owing to its holistic regulatory philosophy and long-standing clinical practice. A significant amount of evidence indicates that bioactive metabolites in TCM have anti-inflammatory, immunomodulatory, and other pharmacological effects, possibly through the modulation of HDACs expression or activity (Wei Y. et al., 2022). It is worth noting that some TCM compounds are expected to reduce adverse reaction risk by selectively regulating specific HDAC isoforms, and thereby effectively alleviate the pathological processes of RA while minimizing interference with normal cellular functions. Therefore, research on the regulatory effects of TCM on HDACs not only facilitates the discovery of safe, effective, and low-cost new anti-RA drugs but also provides a potential path for enhancing treatment outcomes and improving the quality of life for patients.

Based on this, the present paper reviews the mechanisms by which TCM regulates HDACs in RA processes, integrating findings from basic research. The aim is to provide a theoretical foundation for developing novel TCM drugs targeting HDACs and to promote the optimization of precision treatment strategies for RA.

## 2 What are HDACs?

**FIGURE 1:**
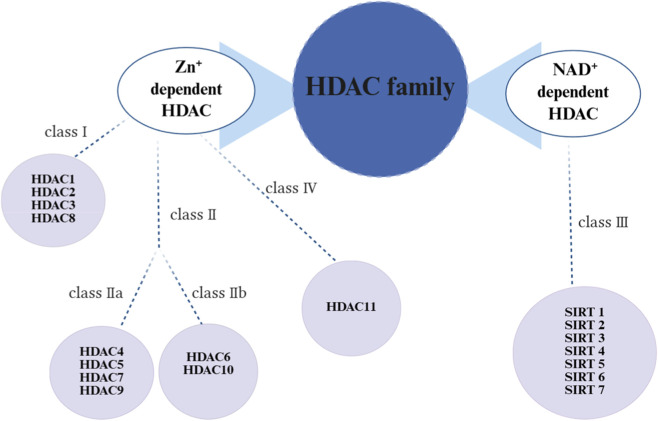
HDAC family classification based on cofactor dependence. The Zn^2+^-dependent branch includes three classes: Class I (comprising HDAC1, HDAC2, HDAC3, HDAC8), Class II (which is subdivided into Class IIa: HDAC4, HDAC5, HDAC7, HDAC9; and Class IIb: HDAC6, HDAC10), and Class IV (comprising only HDAC11). The NAD^+^-dependent branch corresponds to Class III HDAC, which consists of seven members: SIRT1, SIRT2, SIRT3, SIRT4, SIRT5, SIRT6, and SIRT7.

HDACs, as key hubs in the regulatory network of cellular physiological activities, are classified in a variety of ways and are crucial for understanding cellular function. At present, HDACs can be categorized into four classes, Class I, Class II, Class III, and Class IV, according to their sequence homology with the yeast homologous proteins Rpd3, HdaI, and SIR2. From the perspective of dependent cofactors, they can be divided into two categories, “Zn^2+^-dependent” HDACs and “NAD^+^-dependent” HDACs (Wei et al., 2022), which are categorized in the manner illustrated in Figure 1.

“Zn^2+^-dependent” HDACs include 11 isoforms, encompassing Class I, Class IIa, Class IIb, and Class IV (Zheng, 2022). Class I HDACs, are primarily localized in the nucleus and play a key role in regulating cell proliferation and growth. This group comprises HDAC1, HDAC2, HDAC3, and HDAC8, are primarily located within the cell nucleus and are pivotal in the regulation of gene expression, thereby inhibiting gene transcription as core metabolites of epigenetic regulation (Luo and Li, 2020). Class II HDACs exhibit homology with yeast Hda1 and are distinctly categorized into subgroups. Class IIa contains HDAC4, HDAC5, HDAC7, and HDAC9, while Class IIb includes HDAC6 and HDAC10 (Mazzocchi et al., 2020). Class II HDACs exhibit a remarkable ability to shuttle between the nucleus and cytoplasm. Within the nucleus, HDACs remove acetyl groups from histones H3 and H4, leading to chromatin condensation and gene expression repression. Beyond histones, they regulate the acetylation state of non-histone substrates, impacting various biological processes like cell differentiation, proliferation, migration, and apoptosis (Kutil et al., 2022). HDAC11 is the only member of Class IV HDACs, which has some structural commonalities with Class I and II HDACs. However, it exhibits limited amino acid sequence homology and is primarily localized in the cytoplasm and mitochondria (Chen H. et al., 2022).

“NAD^+^-dependent” HDACs constitute Class III, a subgroup with seven isoform members, SIRT1–SIRT7, with NAD^+^ serving as the catalytic cofactor. SIRT1 (Kong et al., 2013), SIRT6 (Wei et al., 2024), and SIRT7 (Kiran et al., 2013) are mainly found in the nucleus. SIRT2 can dynamically shuttle between the nucleus and cytoplasm (Wang et al., 2018). SIRT3, SIRT4, and SIRT5 are chiefly located in the mitochondria (Hussain et al., 2021). The activity of these deacetylases is tightly regulated by cellular NAD^+^ levels, linking energy status to metabolic and epigenetic networks. The dysfunction of sirtuins is closely related to the development of autoimmune diseases (Tao et al., 2023). Their aberrant expression or altered activity affects autoimmune responses by regulating key pathways such as immunoin flammation, oxidative stress, and mitochondrial function.

**FIGURE 2:**
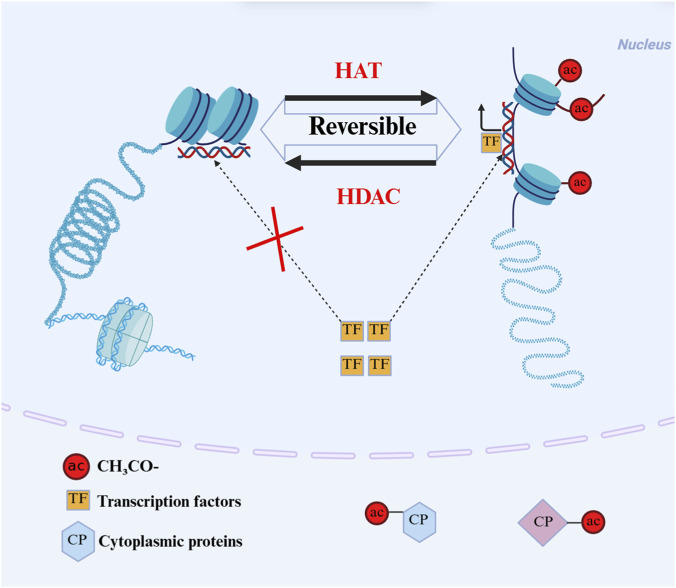
Schematic diagram of reversible regulation of histone acetylation and the role of acetylation in the nucleus and cytoplasm. HAT and HDAC mediate reversible acetylation modification via catalyzing histone acetylation by HAT to loosen chromatin and enable transcription factor binding, driving histone deacetylation by HDAC (with its inhibition sustaining the acetylated state of histones), and regulating cytoplasmic protein functions through acetylation modification. HAT: Histone acetyltransferase; HDAC: Histone deacetylase; ac: Acetylation (the covalent addition of an acetyl group (CH_3_CO-) to histone/non-histone proteins); TF: Transcription factors; CP: Cytoplasmic proteins.

HATs are a class of epigenetic regulatory enzymes that antagonize the function of HDACs. HATs promote gene transcription activation by catalyzing histone acetylation, which promotes the depolymerization of tightly packed heterochromatin into loose euchromatin structures (Sterner and Berger, 2000). HDACs and HATs indirectly control the transcriptional switches of downstream genes by regulating their activity, nucleocytoplasmic localization, or protein interaction capacity, thereby shaping processes such as protein growth, differentiation, migration, and activity (Chueh et al., 2015). They ultimately serve as a key epigenetic regulatory hub for maintaining cellular homeostasis, and the synergy and balance of their functions are critical to cell survival and organismal health (Figure 2).

## 3 Targeting HDACs in RA management

**FIGURE 3:**
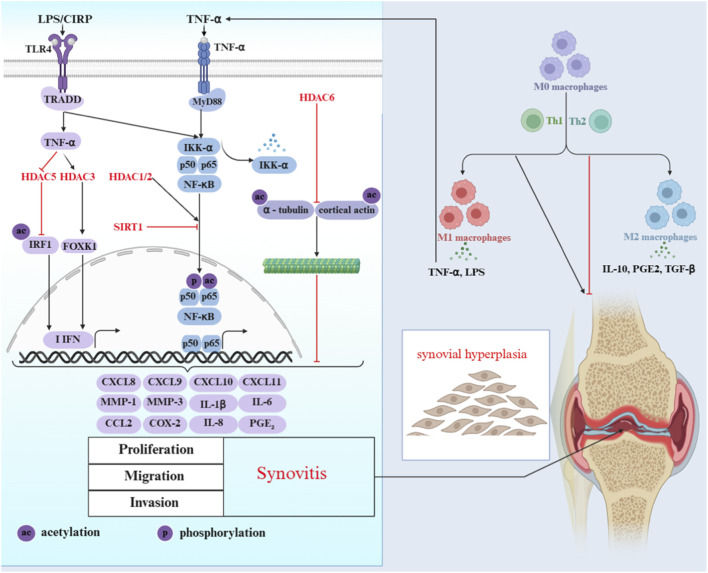
Mechanisms of histone deacetylase and related signaling pathways in the regulation of inflammation in RA synovial. HDAC-related regulatory molecules contribute to synovitis and rheumatoid arthritis joint lesions via modulating the TLR/TNF-α-mediated signaling pathway to promote the secretion of pro-inflammatory factors, regulating the polarization of M0 macrophages toward pro-inflammatory M1 macrophages, and inducing synovial hyperplasia by boosting synovial cell proliferation, migration and invasion. ac: Acetylation; LPS: Lipopolysaccharide; CIRP: Cold-inducible RNA-binding protein; TLR4: Toll-like receptor 4; TRADD: TNF receptor-associated death domain protein; MyD88: Myeloid differentiation primary response 88; IKK-α: Inhibitor of nuclear factor kappa-B kinase alpha; NF-κB: Nuclear factor kappa-light-chain-enhancer of activated B cells; HDAC: Histone deacetylase; SIRT1: Sirtuin 1; IRF1: Interferon regulatory factor 1; FOXK1: Forkhead box K1; IFN: Interferon; CXCL: C-X-C motif chemokine ligand; MMP: Matrix metalloproteinase; IL: Interleukin; CCL2: C-C motif chemokine ligand 2; COX-2: Cyclooxygenase-2; PGE_2_: Prostaglandin E_2_; Th1/Th2: T helper 1/2 cells.

TCM targets HDACs to intervene in RA, which holds great significance for clinical treatment. Its core advantage lies in its ability to precisely regulate key pathological links during the onset and progression of RA by multiple pathways. This process not only regulates the abnormal behavior of inflammatory synovial cells and corrects the dysregulation of immune cell function, but also improves the imbalance of bone metabolism (see Figure 3). The specific mechanism of action will be analyzed in further detail below (Table 1).

**TABLE 1:** Mechanisms by which different HDAC isoforms regulate rheumatoid arthritis.

Mechanism type	HDAC isoforms	Core mechanism	References
Direct Enzymatic Mechanism	HDAC1	Regulates CCR6 expression in CD4^+^ T cells via STAT3-dependent pathway; promotes Th17-associated cytokines (IL-6, IL-17) production; HDAC1 deficiency impairs CCR6 upregulation in IL-6-stimulated CD4^+^ T cells	Göschl et al. (2020)
		Promotes deacetylation of p65 at K122/K123 via interaction with p65; facilitates deacetylation of β-catenin at K49 via binding to β-catenin; regulates nuclear localization and transcriptional activity of p65 and β-catenin	Iezaki et al. (2016)
		Inhibit TNF-α-induced MMP-1 secretion via deacetylation; MMP-1 upregulates after HDAC1 knockdown	Horiuchi et al. (2009)
	HDAC1, HDAC2	Inhibit p16/p21/p53 expression via deacetylation, promote RA-SF proliferation and inhibit apoptosis	
		Inhibits osteoclast formation and bone resorption; downregulates mRNA expression of osteoclast-related genes (NFATc1, TRAF-6, OSCAR) and inflammatory chemokines (MCP-1, MIP-1α) in LPS/TNF-α-stimulated monocytes; acts during early osteoclast differentiation (effective when treatment starts at day 7 post-RANKL stimulation)	Cantley et al. (2015)
	HDAC2	Promotes FLS proliferation, migration and invasion, inhibits FLS apoptosis, and increases secretion of inflammatory factors (TNF-α, IL-1β, IL-6, IL-17)	Mao et al. (2023)
	HDAC3	Suppresses IL-1β-induced inflammatory gene expression (cytokines, MMPs, IFNB1), reduces RA FLS invasive capacity and secretion of inflammatory factors	Angiolilli et al. (2017)
		Promotes proliferation, migration and invasion of RA-FLS; upregulates N-cadherin and MMP-3 expression; enhances secretion of IL-1β and IL-33; its knockdown or inhibition suppresses above abnormal activation of RA-FLS	Yao et al. (2024)
		Interacts with FOXK1 to deacetylate it, maintaining FOXK1 stability and activating interferon signaling; promotes RA-FLS proliferation, migration, invasion, and inhibits apoptosis; propionate disrupts this interaction, increasing FOXK1 acetylation and reducing its stability to block interferon signaling	Chen et al. (2024)
	HDAC4	Inhibits proliferation, migration of RA-FLS, promotes apoptosis; reduces secretion of inflammatory factors (TNF-α, IL-1β, IL-6, IL-8) and chemokines (CXCL1, CXCL5, CXCL8, CCL2); alleviates RA pathological damage	Chang and Kan (2021)
	HDAC5	Inhibits IL-1β-induced transcription of type I interferon response genes (IFNB, CXCL9, CXCL10, CXCL11); regulates nuclear retention of transcription factor IRF1	Angiolilli et al. (2016)
	HDAC6	Increases acetylation of tubulin and cortactin; suppresses RA-FLS migration, invadopodia formation, and secretion of IL-6, MMP-1, MMP-3, CCL2, CXCL8, CXCL10; downregulates ICAM-1/VCAM-1 expression; alleviates synovitis, cartilage damage and bone erosion *in vivo*	Park et al. (2021)
	HDAC1, 2, 3, 8	Decreased mRNA and protein expression; HDAC3 activity is most significantly reduced; nuclear HAT activity is increased; total histone H3 acetylation is elevated; disrupts HDAC/HAT balance	Li Y. et al. (2018)
Upstream Pathway-Mediated Mechanism	HDAC1	T cell-specific HDAC1 deletion renders mice resistant to CIA; MS-275 dose-dependently inhibits CCR6 expression in murine/human Th17 cells; RA patients have elevated HDAC1 expression in synovial CD4+CCR6+ T cells, correlating with disease-related T cell infiltration	Göschl et al. (2020)
		Promotes deacetylation of p65 at K122/K123 via interaction with p65; facilitates deacetylation of β-catenin at K49 via binding to β-catenin; regulates nuclear localization and transcriptional activity of p65 and β-catenin	Iezaki et al. (2016)
	HDAC1, HDAC2	NW-21 and MS-275 reduce paw inflammation, synovial inflammatory cell infiltration, and pannus formation; attenuate bone and cartilage destruction, increase bone volume, and decrease TRAP-positive osteoclasts on bone surfaces	Cantley et al. (2015)
	HDAC2	Exacerbates RA progression by regulating the IL-17-CCL7 signaling pathway	Mao et al. (2023)
​	HDAC3	Regulates IFN-β production to promote STAT1 Tyr701 phosphorylation and downstream chemokine (CXCL9, CXCL11) expression	Angiolilli et al. (2017)
		Extracellular CIRP induces HDAC3 expression via TLR4; blocking CIRP or inhibiting HDAC3 (RGFP966) alleviates synovial hyperplasia, cartilage and bone destruction, and reduces arthritis severity	Yao et al. (2024)
		*Bacteroides fragilis* produces propionate (HDAC3 inhibitor); propionate monotherapy or combination with anti-TNF etanercept alleviates synovial hyperplasia, bone erosion, and cartilage destruction in CIA mice; *B. fragilis* and propionate levels are higher in healthy individuals and CIA-resistant mice	Chen et al. (2024)
	HDAC4	circFBXW7 (delivered by MSC-derived exosomes) sponges miR-216a-3p to upregulate HDAC4 expression, thereby exerting anti-RA effects	Chang and Kan (2021)
	HDAC5	Expression is negatively correlated with RA disease activity (CRP, ESR, DAS28); suppressed by inflammatory cytokines (IL-1β, TNF), and its silencing potentiates inflammation-related gene expression	Angiolilli et al. (2016)
	HDAC6	Activity is upregulated in RA synovial tissue; regulated by inflammatory stimuli (IL-1β); its inhibition blocks NF-κB pathway and reactive oxygen species production, interfering with cytoskeletal reorganization and pro-inflammatory signaling	Park et al. (2021)
	HDAC1, 2, 3, 8	Expression and activity of Class I HDACs are negatively correlated with RA disease activity (CRP, ESR, DAS28); HDAC activity is inversely associated with pro-inflammatory cytokines (TNF-α, IL-6); TSA induces PBMC apoptosis; histone hyperacetylation may promote pro-inflammatory processes and contribute to RA pathogenesis	Li Y. et al. (2018)

CCL, C-C motif chemokine ligand; CCR6, C-C Motif Chemokine Receptor 6; CIA, Collagen-Induced Arthritis; CIRP, Cold-Inducible RNA-Binding Protein; CRP, C-Reactive Protein; CXCL, C-X-C motif chemokine ligand; circFBXW7, Circular RNA, circFBXW7; cortactin, Cortactin; DAS28, Disease Activity Score 28; ESR, erythrocyte sedimentation rate; FLS, Fibroblast-Like Synoviocyte; FOXK1, Forkhead Box K; HAT, histone acetyltransferase; HDAC, histone deacetylase; ICAM-1, Intercellular Adhesion Molecule-1; IFN-β, interferon beta; IFNB1, Interferon Beta 1; IL-1β, Interleukin-1, beta; IL-, Interleukin-; IRF1, Interferon Regulatory Factor 1; K122/K123, Lysine 122/Lysine 123; K49, Lysine 49; LPS, lipopolysaccharide; MAPK, Mitogen-Activated Protein Kinase; MCP-1, Monocyte Chemoattractant Protein-1; MIP-1α, Macrophage Inflammatory Protein-1α; MMP, matrix metalloproteinase; MS-275, Class I Histone Deacetylase Inhibitor; MSC, mesenchymal stem cell; miR-216a-3p, microRNA-216a-3p; NFATc1, Nuclear Factor of Activated T Cells 1; NFκB, Nuclear Factor Kappa-B; N-cadherin, N-Cadherin; NW-21, HDAC1-Targeting Inhibitor NW-21; OSCAR, Osteoclast-Associated Receptor; p16/p21/p53, Cyclin-Dependent Kinase Inhibitor p16/p21 and Tumor Suppressor p53; p38, p38 Mitogen-Activated Protein Kinase; p65, Nuclear Factor Kappa-B p65 Subunit; PBMC, peripheral blood mononuclear cell; RA, rheumatoid arthritis; RA-SF, rheumatoid arthritis synoviocytes; RANKL, Receptor Activator of Nuclear Factor Kappa-B ligand; RGFP966, HDAC3 inhibitor; SCFAs, Short-Chain Fatty Acids; STAT, signal transducer and activator of transcription; TLR4, Toll-Like Receptor 4; TNF-α, Tumor Necrosis Factor-α; TRAF-6, Tumor Necrosis Factor Receptor-Associated Factor 6; TRAP, Tartrate-Resistant Acid Phosphatase; TSA, Trichostatin A; tubulin, Tubulin; Tyr701, Tyrosine 701; VCAM-1, Vascular Cell Adhesion Molecule-1; β-catenin, Beta-Catenin.

### 3.1 Mechanisms to inhibit proliferation and invasion of inflammatory synovial cells

In RA pathology, FLS are key pathogenic cells that show proliferative and invasive phenotypes, secrete proteases and pro-inflammatory cytokines to damage cartilage and bone, are divided into multiple functional subtypes based on surface markers, possess phenotypic plasticity, and maintain inflammatory memory by interacting with T cells, B cells, and macrophages (Wu Z. et al., 2021). These invasive FLS secrete cytokines, MMPs, and other substances that promote inflammation and invade cartilage and bone tissue. This pathogenesis is tightly regulated by Zn^2+^-dependent HDACs and NAD^+^-dependent HDACs.

#### 3.1.1 Zn^2+^ dependent HDACs

In the complex pathological process of RA, Zn^2+^-dependent HDACs play a crucial and multifaceted role. It is worth noting that different isoforms of the Zn^2+^-dependent HDAC family exhibit distinct functional differences during the abnormal activation of RA synovial cells and the progression of inflammation. Some isoforms display potential anti-inflammatory properties. For instance, HDAC4 exhibits significant downregulation in synovial tissue derived from patients suffering from RA. Research findings indicate that mesenchymal stem cell-derived exosomal circFBXW7 is capable of increasing HDAC4 expression through the sequestration of miR-216a-3p. This mechanism successfully restrains the proliferation and inflammatory reactions of RA-FLS, markedly reduces joint injury in RA model rats, and clearly exhibits the anti-inflammatory properties of HDAC4 (Chang and Kan, 2021). Similarly, the mRNA expression of HDAC5 in the synovial tissue of RA patients was negatively correlated with important activity parameters, such as erythrocyte sedimentation rate and disease activity score 28. Moreover, inflammatory factors such as IL-1β and TNF can selectively inhibit the expression of HDAC5, and the deletion of HDAC5 will lead to the accumulation of transcription factor IRF1 in the nucleus, enhancing the transcription of type I interferon response genes and further highlighting the anti-inflammatory function of HDAC5 (Angiolilli et al., 2016).

However, certain isoforms exhibit pro-inflammatory effects. HDAC1 expression levels in RA-SFs is significantly heightened compared to those in osteoarthritis synovial fibroblasts. When HDAC1 is knocked down, the cell count and proliferation of RA-SF decrease, while apoptosis increases, and TNF-α-induced MMP-1 production is upregulated, indicating that HDAC1 contributes inflammatory process by promoting the proliferation and survival of RA-SF (Horiuchi et al., 2009). HDAC2 exhibits significant upregulation in the synovial tissues from RA patients and with CIA rats. The overexpression of HDAC2 enhances the proliferation, migration, and invasion of FLS, inhibits apoptosis, increases the secretion of inflammatory factors, and exacerbates RA progression through modulating the IL-17-CCL7 signaling pathway, showing its pro-inflammatory properties (Mao et al., 2023). The expression of HDAC3 in synovial tissue from RA patients is markedly elevated, and extracellular cold-inducible RNA-binding protein could upregulate the expression of HDAC3 through TLR4, inducing abnormal activation of RA- FLS. HDAC3 inhibitors can effectively alleviate the severity of arthritis in AA rats (Yao et al., 2024). Additionally, HDAC3 also participates in the regulation of inflammatory gene expression in RA FLS by regulating the production of type I interferon and facilitating the activation of STAT1 (Angiolilli et al., 2017). At the same time, it interacts with FOXK1 to maintain the FOXK1 stability, enhance the activity of interferon signaling pathways, and induce abnormal activation of RA-FLS. However, propionate produced by bacteroidetes fragilis can disrupt this interaction, reduce the stability of FOXK1, block interferon signaling, and thus inhibit the abnormal activation of RA-FLS (Chen et al., 2024). HDAC6 overexpression catalyzes the deacetylation of α-tubulin and cortical actin, disrupting the stability of the cytoskeleton. This process promotes reorganization of the actin cytoskeleton, thereby boosting the capacity of RA-FLS for migration and invasion. Additionally, HDAC6 upregulates the expression of MMPs and also promotes the expression of inflammatory factors and chemokines including CCL2, CXCL8, and CXCL10 (Park et al., 2021). Furthermore, HDAC6 activates CMA by upregulating Hsc70 and LAMP2A, thereby promoting the progression of RA. This process eliminates misfolded proteins, alleviates cytotoxic stress, and sustains the proliferation and survival of abnormal RA-FLS. Targeted inhibition of HDAC6 (via CAY10603 or shRNA) downregulates Hsc70 and LAMP2A, suppresses CMA activity, reduces RA-FLS proliferation and TNF-α/IL-6 secretion, and alleviates synovial inflammation and cartilage/bone damage in CIA mice (Lin et al., 2024). These effects exacerbate the inflammatory response, indicating that HDAC6 have pro-inflammatory and pro-tissue destruction effects.

#### 3.1.2 NAD^+^-dependent HDACs

Zn^2+^-dependent HDACs use Zn^2+^ for their catalytic activity and have specific roles in promoting or reducing inflammation based on their isoform. In contrast, NAD^+^-dependent HDACs need NAD^+^ as a cofactor, connecting their activity to the cell’s energy metabolism. SIRT1 is a major NAD^+^-dependent deacetylase. It uses NAD^+^ to deacetylate substrates by combining this modification with the breakdown of NAD^+^. This process plays a crucial role in managing metabolism, reducing oxidative stress, promoting mitochondrial creation, and controlling inflammation (Langley and Sauve, 2013). SIRT1 exerts a versatile role in sustaining cellular homeostasis among these enzymes. Its activity is modulated by intracellular NAD^+^ levels, energy stress, and protein-protein interactions.

In RA, SIRT1 shows cell-type-dependent expression and dual regulatory effects. The level of SIRT1 expression is markedly reduced in the FLS cells of individuals with RA. At this time, overexpression of SIRT1 can inhibit the NF-κB pathway, which is manifested by decreased expression, phosphorylation, and acetylation of p65. This further exerts an inhibitory role on FLS proliferation, migration, and invasion, promotes FLS apoptosis, and diminishes the secretion of pro-inflammatory cytokines such as TNF-α and IL-6 (Li G. et al., 2018). In contrast, SIRT1 is constitutively highly expressed in monocytes/macrophages in RA synovial tissue and can be further induced by TNF-α. In this case, SIRT1 activates the NF-κB signaling pathway, in turn boosting the production of pro-inflammatory cytokines like IL-6 and IL-8, which exacerbates the local inflammatory reaction. In RA endothelial cells, reduced SIRT1 expression and activity can lead to a series of adverse outcomes, inducing proliferation, pro-apoptotic and pro-angiogenic phenotypes by increasing p53 and p65 acetylation. SIRT1 overexpression can reverse this pathological feature, reduce synovial angiogenesis, and shorten the duration of arthritis (Leblond et al., 2020). In addition, SIRT1 can also construct a “metabolic-immunofeedback” mechanism by regulating glycolysis and lipid metabolism, interacting with AMPK, PPAR-γ, etc., inhibiting HIF-1α in RA-FLS, reducing VEGF secretion, and thereby inhibiting angiogenesis (Wu Y. et al., 2021).

### 3.2 Strategies to enhance immune cell functionality in RA

Abnormal activation of the immune system and imbalance in immune cell function are key factors driving the onset and persistent progression of RA. These factors are closely associated with alterations in epigenetic modifications, particularly the regulation of HDACs. HDACs participate in regulating immune cell function and maintaining immune homeostasis through their effects on conformation, transcription factor activity, and downstream inflammation-related gene expression, which renders them a crucial potential target for RA treatment.

#### 3.2.1 Macrophages

In patients with RA, a significant number of macrophages are found in peripheral blood, synovial fluid, and synovial tissue during the early stages of the disease. It has been found that alterations in HDAC activity are capable of affecting macrophage polarization, inducing a shift in macrophages from the pro-inflammatory M1 phenotype to the anti-inflammatory M2 phenotype (Bhat et al., 2024). HDACi can inhibit inflammatory activation by targeting class I/class II HDACs or class III sirtuin HDACs, reduce the generation of pro-inflammatory cytokines in synovial macrophages of RA patients, induce macrophage apoptosis, and selectively downregulate the expression levels of anti-apoptotic proteins (Grabiec et al., 2012). In addition to the potential demonstrated by HDACi in the treatment of RA, HDAC activators, as an emerging therapeutic strategy, have also gradually come to the fore and brought new hope for RA treatment.

#### 3.2.2 B cells

In the synovial tissue of RA patients, the accumulation of B cells in these tissues is associated with disease severity. Specific B cell subsets are involved in pathological processes such as bone destruction by secreting receptor-activator of nuclear factor-kappaB ligand (RANKL). Cytokines including B cell-activating factor, whereas some B cell subsets exert a protective effect (Wu F. et al., 2021). HDACs regulates regulatory B cell differentiation by inhibiting histone deacetylase activity (Zou et al., 2021). Naive unactivated B cells exhibit hypermethylation and histone deacetylation of genes involved in the B cell lineage. Prior to differentiation, Prdm1 expression is inhibited by Bcl-6. Upon dissociation of Bcl-6 from HDAC, acetylation of the Prdm1 promoter is increased, leading to the induction of Blimp-1 expression. Blimp-1 inhibits Bcl-6 and Pax5 through histone deacetylation of their promoter regions, thereby promoting plasma cell differentiation and antibody production (Srivastava and Rasool, 2024). Understanding how various active metabolites regulate the mechanisms by which HDACs affect B cell activation, differentiation, and antibody production may provide a novel immunomodulatory treatment option for RA.

#### 3.2.3 T cells

In patients with RA, CD4^+^ T cells exhibit hyperacetylation of histone H3 and hypoacetylation of histone H4. Hyperacetylation of H3 promotes the transcription of pro-inflammatory genes, whereas hypoacetylation of H4 suppresses the expression of anti-inflammatory genes (Wu, 2010). Meanwhile, the pathogenesis of RA is closely associated with Th17/Treg imbalance (Niu et al., 2012). The differentiation of Th17 and Treg cells is determined by IL-6 signaling and the activation of STAT3 (Zhou et al., 2008). HDAC1 sustains the expression of the chemokine CCR6 in Th17 cells, promoting their migration to the joint synovium and enhancing the secretion of pro-inflammatory factors to exert a pro-inflammatory effect (Göschl et al., 2020). HDACi increases the acetylation level of FOXP3 in Tregs by inhibiting HDAC activity, thereby enhancing the immunosuppressive function of Tregs and promoting their proliferation (Saouaf et al., 2009). However, not all HDACi act as immunosuppressants. SIRT1, a critical immunosuppressive factor, modulates the inflammatory response in chondrocytes and synovial fibroblasts by suppressing the activity of AP-1 and NF-κB in T cells (Kong et al., 2013).

#### 3.2.4 PBMCs

**FIGURE 4:**
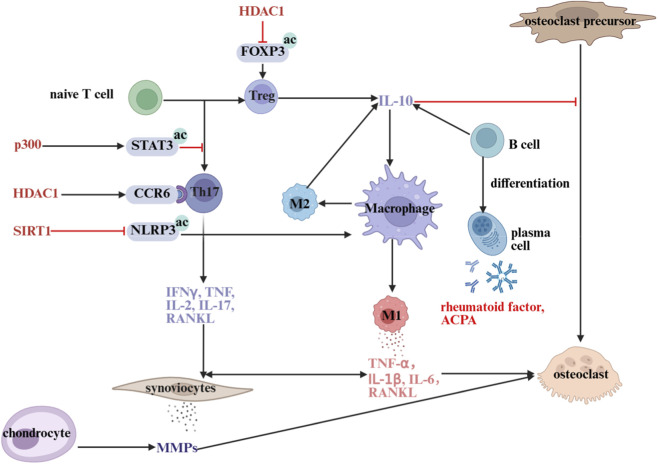
Schematic diagram of HDACs regulating immune cell differentiation mechanism. HDAC promotes inflammation and bone destruction in rheumatoid arthritis via regulating T cell differentiation (modulating Treg/Th17 balance to alter IL-10 and pro-inflammatory cytokine secretion), controlling macrophage polarization (driving M1 polarization to release pro-inflammatory factors), facilitating B cell differentiation into plasma cells to produce autoantibodies (e.g., rheumatoid factor, ACPA), and inducing synoviocytes and chondrocytes to secrete inflammatory factors and MMPs that promote osteoclast formation. ac: Acetylation; HDAC1: Histone deacetylase 1; FOXP3: Forkhead box P3; Treg: Regulatory T cell; Th17: T helper 17 cell; p300: E1A-binding protein p300; STAT3: Signal transducer and activator of transcription 3; CCR6: C-C motif chemokine receptor 6; SIRT1: Sirtuin 1; NLRP3: NOD-like receptor family pyrin domain containing 3; IFNγ: Interferon gamma; TNF: Tumor necrosis factor; IL: Interleukin; RANKL: Receptor activator of nuclear factor kappa-B ligand; MMPs: Matrix metalloproteinases; ACPA: Anti-citrullinated protein antibody.

PBMCs are a heterogeneous population of immune-competent cells in peripheral blood, including monocytes, T cells, B cells, and other cell types. Studies indicate that HDAC3 activity in PBMCs isolated from RA patients is significantly reduced, resulting in elevated histone H3 acetylation levels. This imbalance in HDAC and HAT activity could play a role in RA pathogenesis by activating the expression of pro-inflammatory cytokine-related genes (Li Y. et al., 2018). Figure 4 integrates the interactions between these epigenetic regulators and cells such as immune cells, chondrocytes, and osteoclasts, and intuitively illustrates the cascade regulatory network that drives the progression from abnormal immune responses to joint tissue damage during RA pathogenesis. This provides a reference for further elucidating the disease mechanism and identifying molecular targets for TCM intervention.

### 3.3 Approaches to rectify imbalanced bone metabolism in RA

**FIGURE 5:**
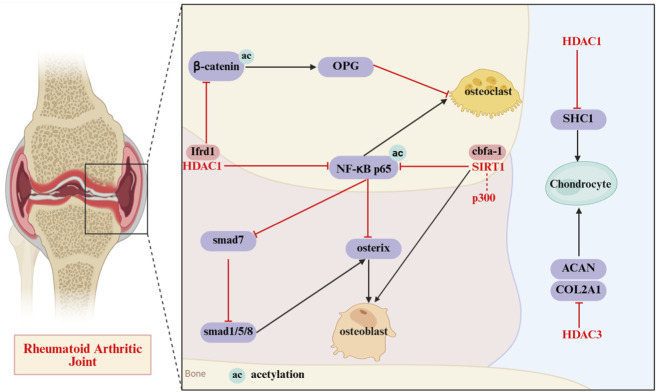
Schematic diagram of the mechanism of HDACs involved in joint osteoblasts, osteoclasts and chondrocytes. HDAC disrupts bone and cartilage homeostasis in rheumatoid arthritis joints via regulating the β-catenin/OPG pathway to affect osteoclast activity, modulating NF-κB-related signals (involving molecules like Irfd1 and the smad family) to control osteoblast differentiation, and regulating the expression of ACAN and COL2A1 in chondrocytes to impair cartilage function. ac: Acetylation; HDAC: Histone deacetylase; β-catenin: Beta-catenin; OPG: Osteoprotegerin; Irfd1: Interferon-related developmental regulator 1; NF-κB p65: Nuclear factor kappa-light-chain-enhancer of activated B cells p65; smad: Sma and Mad related family; SIRT1: Sirtuin 1; cbfa-1: Core-binding factor alpha 1; SHC1: Src homology 2 domain-containing transforming protein 1; ACAN: Aggrecan; COL2A1: Collagen type II alpha 1 chain.

In RA, joint destruction is primarily driven by the functional dysregulation and imbalance among osteoclasts, osteoblasts, and chondrocytes (Fardellone et al., 2014). Abnormal activation of HDACs impairs chondrocytes, disrupting the balance of ECM synthesis and degradation and eventually causing cartilage dysfunction (Komatsu and Takayanagi, 2022). The imbalance in functional coordination between osteoclasts and osteoblasts leads to the dominance of bone resorption. Their differentiation and activity are regulated by the epigenetic mechanisms of HDACs, as shown in Figure 5.

#### 3.3.1 Osteoclasts and osteoblasts

In the inflammatory microenvironment of RA, the dysregulation between osteoclasts and osteoblasts disrupts bone homeostasis, resulting in irreversible progressive bone loss. In RA patients, osteoclast differentiation is regulated by the RANKL/RANK/OPG pathway. HATs enhance RANKL transcription by acetylating its promoter region, which activates downstream signaling cascades and regulates molecules such as nuclear factor of activated T cell c1 (NFATc1) and NF-κB to promote bone resorption. Conversely, histone acetylation of OPG can inhibit excessive osteoclast differentiation (Yu et al., 2024). Studies have found that HDACi can increase the acetylation levels at histone H3K9 and H4K8 sites, and regulate the acetylation status of the kruppel-like factor 2 transcription factor. This regulation inhibits the expression of the autophagy-related gene, thereby promoting osteoclast differentiation and activity (Cantley et al., 2015). The regulation of osteoblast gene expression is controlled by transcription factors such as Runx2 and Osterix, while histone modification in osteoblasts is regulated by epigenetic cofactors including HATs and HDACs. The induction, proliferation, differentiation, and maturation of osteoblasts are precisely controlled by these regulatory mechanisms. In osteoblasts, HDAC binds to Interferon-Related Developmental Regulator 1, which promotes the deacetylation of p65 and β-catenin. This process maintains NF-κB activity to inhibit smad1/5/8-mediated osteogenic differentiation; simultaneously, it reduces the nuclear accumulation of β-catenin, leading to decreased OPG expression. Collectively, these effects inhibit bone formation and promote bone resorption (Iezaki et al., 2016).

#### 3.3.2 Chondrocytes

In an environment enriched with RA-related cells and cytokines, chondrocytes become activated. Normally, the balanced synthesis and degradation of collagen and proteoglycans in the cartilage extracellular matrix maintain proper cartilage function. However, the abnormal activation of HDACs induces the deacetylation of MMP genes, reducing their transcriptional activity. Specifically, HDAC3 inhibits the transcription of COL2A1 and ACAN genes via histone deacetylation. Moreover, it induces the activation of transcription factors like NF-κB and AP-1, leading to enhanced expression of matrix-degrading enzymes such as MMP-13 and ADAMTS-5. This cascade effect results in decreased cartilage matrix synthesis paired with increased degradation, ultimately accelerating the process of cartilage erosion (Meng et al., 2018). HDAC6 modulates the expression of MMPs and inflammatory genes triggered by IL-1 in chondrocytes through regulating the NF-κB signaling pathway. HDAC6 deletion or inhibition boosts the cytoplasmic stability of IκBα, which in turn prevents the nuclear translocation of the NF-κB subunit p65 and the activation of reporter genes. Consequently, this process downregulates the transcription of relevant MMPs and pro-inflammatory factors, thereby reducing both cartilage matrix degradation and inflammatory responses (Park et al., 2021).

## 4 Inhibitors of pro-inflammatory HDAC isoforms

TCM-derived metabolites have garnered significant interest in developing inhibitors targeting pro-inflammatory HDAC isoforms due to their natural, structure-based efficacy. Notably, many metabolites are widely distributed in medicinal plants and not unique to TCM. These metabolites achieve isoform selectivity through structural diversity, providing multiple interaction points with HDAC isoforms. Among them, the cap group binding to the HDAC active site surface and the ZBG chelating Zn^2+^ are key components (Li et al., 2022). Many TCM metabolites inherently possess these structural domains, allowing them to interact precisely with HDACs via non-covalent interactions and chelation, disrupting the enzyme’s ability to hydrolyze acetyllysine. This structural adaptation elucidates TCM’s anti-inflammatory mechanisms and acts as a template for developing selective HDAC inhibitors with low toxicity. Additionally, TCM metabolites can engage multiple targets, modulating complex signaling pathways, and stabilizing specific HDAC conformations (Table 2). Over time, they may naturally evolve selectivity patterns and act as allosteric modulators, inducing isoform-specific conformational changes, offering unique advantages over synthetic HDAC modulators.

**TABLE 2:** The anti-RA effects and mechanisms of TCM inhibitors.

Name	Compound class	Latin binomial	Experimental type	Model	Dosages tested (Route of administration)	Key outcomes (effective dose)	Safety signals	References
Emodin	anthraquinone	-	*ex vivo* human	Human (RA patient-derived synoviocytes), n = 3 (17, 28, 60 years old), IL-1β (10 ng/mL) + LPS (100 ng/mL) under hypoxia (1% O_2_, 5% CO_2_, 95% N_2_)	0, 0.01, 0.1, 1, 10, 100 μM (cell proliferation assay); 0, 1, 10 μM (HDAC activity assay)	RA synoviocyte proliferation (IL-1β+LPS-induced) ↓; TNF-α; IL-6; IL-8↓; PGE_2_; MMP-1; MMP-13↓; VEGF↓; mRNA (COX-2; VEGF; HIF-1α; MMP-1; MMP-13) ↓; Total HDAC activity↓; HDAC1↓ (HDAC2: no change); NF-κB DNA-binding activity; p-ERK/p-p38: no change	No cytotoxicity in RAW264.7 cells and HUVECs	Ha et al. (2011)
Ellagic Acid	polyphenol	-	*in vitro*	Human (MH7A), n = 3 replicate experiments, TNF-α (10 ng/mL) stimulation for 24 h	0, 10, 25, 50, 100 μM (50 μM for main experiments)	MH7A cell proliferation↓; apoptosis↑; IL-6; IL-1β; TNF-α↓; MDA↓; SOD↑; MTA1↓; HDAC1↓; Nur77↑	100 μM impairs MH7A cell viability; lower doses (10–50 μM) have no obvious cytotoxicity	Song et al. (2021)
			*in vivo*	Wistar rats, n = 8 per group, intradermal injection of bovine type II collagen + complete Freund’s adjuvant emulsion (immunization on day 0, booster on day 7)	25, 50, 100 mg/kg (oral gavage, once daily from day 21 to day 42)	Arthritis index↓; paw swelling↓; synovial hyperplasia↓; inflammatory cell infiltration↓; TNF-α; IL-6; IL-1β↓; MTA1↓; HDAC1↓; Nur77↑ (50 mg/kg shows optimal efficacy)	No adverse reactions observed	Song et al. (2021)
Geniposide	Iridoid glycoside	-	*in vitro*	Human (HUVECs), n = 3 replicate experiments, induced by SphK1 activator K6PC-5 (10 µM)	0, 25, 50, 100 µM	SphK1/p-SphK1; S1PR1; p-PI3K/p-Akt; HDAC3/6; HIF-1α↓; ac-HIF-1α↑; VEGF/VEGFR2 (protein + mRNA)↓; HUVECs proliferation; migration; tube-forming ability↓; S1P secretion↓	No adverse reactions observed	Li (2025)
			*in vivo*	SD rats, n = 8 per group, CIA (primary immunization on day 0, booster on day 7)	0, 30, 60, 120 mg/kg (oral gavage, once daily for 2 weeks)	Arthritis index; paw swelling; joint surface temperature↓; synovial hyperplasia; inflammatory infiltration; neovascularization (Vimentin/NG2/CD31)↓; serum S1P; VEGF↓; synovial SphK1/p-SphK1; S1PR1; HDAC3/6; HIF-1α; VEGF/VEGFR2↓; ac-HIF-1α↑	No adverse reactions observed	Li (2025)
*Ephedra sinica* polysaccharide	Polysaccharide	*Ephedra sinica* Stapf	*in vitro*	Mouse (FLS cell line), n = 3 replicate experiments; pretreated with 100 nM IL-1β + 100 nM TNF-α for 24 h in partial experiments	0, 5, 10, 20 μg/mL	FLS proliferation; migration↓; TLR4; MyD88; TRAF6↓; NF-κB p65↓; HDAC1; HDAC2↓	No adverse reactions observed	Ma et al. (2024)
			*in vivo*	C57BL/6J mice, n = not specified, induced by bovine type II collagen + complete Freund’s adjuvant (primary immunization on day 0, booster on day 7)	0, 200, 400 mg/kg (oral gavage, once daily for 21 days)	Arthritis index; paw swelling↓; synovial inflammatory infiltration; cartilage damage↓; serum IL-1β; IL-6↓; intestinal barrier function restored (ZO-1↑); gut microbiota imbalance improved (anti-inflammatory bacteria↑; pro-inflammatory bacteria↓); SCFAs (butyric acid↑; propionic acid↓); serum metabolites (L-tyrosine; sn-glycero-3-phosphocholine↑; N-palmitoyl taurine↓)	High-dose does not affect spleen/thymus weight	Ma et al. (2024)
Guizhi-Shaoyao-Zhimu Decoction	-	*Cinnamomum cassia* Presl *Paeonia lactiflora* Pall *Glycyrrhiza uralensis* Fisch *Ephedra sinica* Stapf *Zingiber officinale* Roscoe *Atractylodes macrocephala* Koidz *Anemarrhena asphodeloides* Bunge *Saposhnikovia divaricata* (Turcz.) Schischk *Aconitum carmichaelii* Debeaux	*in vitro*	Human (HFLS-RA), n = 3 replicate experiments, induced by IL-1β (10 ng/mL)	5.12 × 10^−5^; 2.56 × 10^−4^; 1.28 × 10^−3^ μg/mL (incubated for 24 h)	HDAC1; HSP90AA1; NFKB2; IKBKB; TNF-α↓	No significant side effects observed in long-term clinical trials	Guo et al. (2016)
			*in vivo*	Lewis rats, n = Normal (12), Model (20), low/middle/high (8 per group), MTX (8), induced by intradermal injection of Freund’s CFA at tail base (0.1 mL)	4.65; 9.3; 18.6 g/kg/day (oral gavage, once daily for 21 days)	Arthritis index; incidence; percentage of arthritic limbs↓; joint redness/swelling; synovial inflammation; cartilage/bone destruction↓; HDAC1; HSP90AA1; NFKB2; IKBKB; TNF-α↓(dose-dependent)	No significant side effects observed in long-term clinical trials	Guo et al. (2016)
Fengshining Decoction	-	*Notopterygium incisum* Ting ex H.T.Chang *Angelica pubescens* Maxim. f. *biserrata* Shan et Yuan *Sinomenium acutum* (Thunb.) Rehder and E.H.Wilson *Clematis chinensis* Osbeck *Curcuma zedoaria (Christm.)* Roscoe *Saposhnikovia divaricata* (Turcz.) Schischk *Ligusticum chuanxiong* Hort *Ephedra sinica* Stapf *Cinnamomum cassia* Presl *Sparganium stoloniferum* (Graebn.) Buch. - Ham. ex Juz *Dracaena cochinchinensis* (Lour.) S.C.Chen *Corydalis yanhusuo* (Y.H.Chou and C.C.Hsu) W.T.Wang ex Z.Y.Su and C.Y.Wu *Achyranthes bidentata* Blume *Rehmannia glutinosa* (Gaertn.) DC. *Amomum villosum* Lour	*in vitro*	Mouse (FLS cell line), n = 3 replicate experiments, induced by IL-1β (100 nM) + TNF-α (100 nM) for 24 h	0%, 2.5%, 5%, 10%, 20%, 40% FSN-containing serum (incubated for 24, 48, 72 h)	FLS proliferation; migration↓; apoptosis↑; HDAC1; HDAC2↓; NF-κB p65/P-p65↓; IL-1β; IL-6↓; F-actin stress fiber reorganization inhibited	No significant side effects observed	Wen et al. (2025)
			*in vivo*	C57BL/6J mice, n = not specified per group, induced by bovine type II collagen + complete Freund’s adjuvant (primary immunization on day 0, booster on day 7)	20, 40, 80 g/kg/day (oral gavage, once daily for 21 days)	Arthritis index; paw swelling↓; synovial inflammation; cartilage/bone destruction↓; intestinal barrier function restored (ZO-1; Occludin; MUC2↑); gut microbiota imbalance improved (SCFA-producing bacteria↑; pathogenic bacteria↓); HDAC1/2; NF-κB p65/P-p65↓; serum IL-1β; IL-6↓; butyrate level↑	No significant side effects observed	Wen et al. (2025)
Wutou Decoction	-	*Ephedra sinica* Stapf *Aconitum carmichaelii* Debeaux *Glycyrrhiza uralensis* Fisch *Paeonia lactiflora* Pall *Astragalus mongholicus* Bunge	*in vitro*	Human (MH7A), n = 3 replicate experiments, induced by TNFα (40 ng/mL) stimulation for 24 h	Low: 1 mg/mL, High: 10 mg/mL (*in vitro* culture, 24–48 h treatment); Triptolide 20–40 nM	FLS proliferation; migration; invasion↓; FLS-induced HUVEC tube formation↓; HDAC7 (mRNA + protein) ↓; no obvious effect on FLS apoptosis	No obvious cytotoxicity at tested concentrations	Liu P. et al. (2025)
			*in vivo*	Wistar rats, n = 10 per group, induced by collagen-induced arthritis (CIA: bovine type II collagen + complete Freund’s adjuvant, primary + booster immunization)	Low: 3.75 g/(kg·d), High: 7.5 g/(kg·d)(oral gavage, once daily for 28 days)	Arthritis score; joint swelling↓; synovial hyperplasia; synovial angiogenesis↓; CD31; vWF (vascular markers) ↓; synovial HDAC7 expression↓; synovial lining layer thickness↓	No obvious toxicity; No significant organ damage reported	Liu P. et al. (2025)
			*in vivo*	Wistar rats, n = 6 per group, induced by complete Freund’s adjuvant (CFA, 0.1 mL containing 10 mg/mL dead *Mycobacterium tuberculosis*) via hind footpad injection	9.8 g/kg/day (oral gavage, once daily for 15 days)	Hind paw swelling↓; articular cartilage damage↓; cartilage thickness recovery↑; synovial inflammation↓; LOC101928120 (mRNA)↑; SHC1 (mRNA + protein) ↓; Ahr nuclear translocation↑	No obvious toxicity reported	Wu D. et al. (2021)
			*in vitro*	Human chondrocyte cell line (CHON-001), n = 3 replicate experiments, induced by IL-1β (10 ng/mL) stimulation for 24 h	1 μg/mL (*in vitro* culture); Ras inhibitor BI-3406 (1 μM); siRNA (SHC1/LOC101928120) for mechanism validation	Cell viability↑; apoptosis↓; ROS production↓; aggrecan; COL2A1 (mRNA)↑; MMP-13 (mRNA)↓; Ahr activation (nuclear translocation) ↑; LOC101928120↑; SHC1 (mRNA + protein) ↓; LOC101928120 recruits HDAC1 to SHC1 promoter → histone 3 deacetylation↑	No obvious cytotoxicity at tested concentration	Wu D. et al. (2021)

↑ = Increase/Promote/Upregulate; ↓ = Decrease/Inhibit/Downregulate; ac-HIF-1α, Acetylated Hypoxia-Inducible Factor-1α; Ahr, Aryl Hydrocarbon Receptor; Ahr, Aryl Hydrocarbon Receptor; Akt, Protein Kinase B; BI-3406, Ras Inhibitor BI-3406; BrdU, 5-Bromo-2′-deoxyuridine; C57BL/6J mice, C57BL/6J Mouse Strain; CHON-001, Human Chondrocyte Cell Line CHON-001; CFA, Complete Freund’s Adjuvant; COL2A1, Collagen Type II, Alpha 1 Chain; ELISA, Enzyme-Linked Immunosorbent Assay; ERK, Extracellular Signal-Regulated Kinase; F-actin, Filamentous Actin; FLS, Fibroblast-Like Synoviocyte; HFLS-RA, Human Fibroblast-Like Synoviocyte from Rheumatoid Arthritis; HDAC, histone deacetylase; HIF-1α, Hypoxia-Inducible Factor-1α; HUVECs, Human Umbilical Vein Endothelial Cells; IKBKB, Inhibitor of Nuclear Factor Kappa B Kinase Subunit Beta; IL-1β, Interleukin-1, beta; LPS, lipopolysaccharide; Lewis rats, Lewis Rat Strain; LOC101928120, Long Non-Coding RNA LOC101928120; MMP, matrix metalloproteinase; MMP-1, Matrix Metalloproteinase-1; MMP-13, Matrix Metalloproteinase-13; MDA, malondialdehyde; MH7A, Human Fibroblast-Like Synoviocyte Cell Line MH7A; MTA1, Metastasis-Associated Protein 1; MTX, methotrexate; MUC2, Mucin 2; MyD88, Myeloid Differentiation Primary Response 88; NF-κB, Nuclear Factor Kappa-B; NFKB2, Nuclear Factor Kappa B Subunit 2; Nur77, Nuclear Receptor Subfamily 4 Group A Member 1; Occludin, Occludin; p-Akt, Phosphorylated Protein Kinase B; p-p65, Phosphorylated Nuclear Factor Kappa-B p65 Subunit; P-p65, Phosphorylated Nuclear Factor Kappa-B p65 Subunit; p-PI3K, Phosphorylated Phosphoinositide 3-Kinase; p-SphK1, Phosphorylated Sphingosine Kinase 1; p38, p38 Mitogen-Activated Protein Kinase; PI3K, Phosphoinositide 3-Kinase; PGE_2_, Prostaglandin E_2_; RAW264.7, Mouse Macrophage Cell Line RAW264.7; RA, rheumatoid arthritis; RT-PCR, Reverse Transcription-Polymerase Chain Reaction; SCFAs, Short-Chain Fatty Acids; SHC1, SHC, Adaptor Protein 1; siRNA, Small Interfering RNA; S1PR1, Sphingosine-1-Phosphate Receptor 1; SphK1, Sphingosine Kinase 1; SOD, superoxide dismutase; SD, rats, Sprague-Dawley Rat Strain; TRAF6, Tumor Necrosis Factor Receptor-Associated Factor 6; TLR4, Toll-Like Receptor 4; TNF-α, Tumor Necrosis Factor-α; VEGFR2, Vascular Endothelial Growth Factor Receptor 2; VEGF, vascular endothelial growth factor; Western blot, Western blotting; Wistar rats, Wistar Rat Strain; ZO-1, Zonula Occludens-1; K6PC-5, SphK1 Activator K6PC-5; MMP-1, Matrix Metalloproteinase-1; MMP-13, Matrix Metalloproteinase-13.

### 4.1 TCM bioactive compounds

**FIGURE 6:**
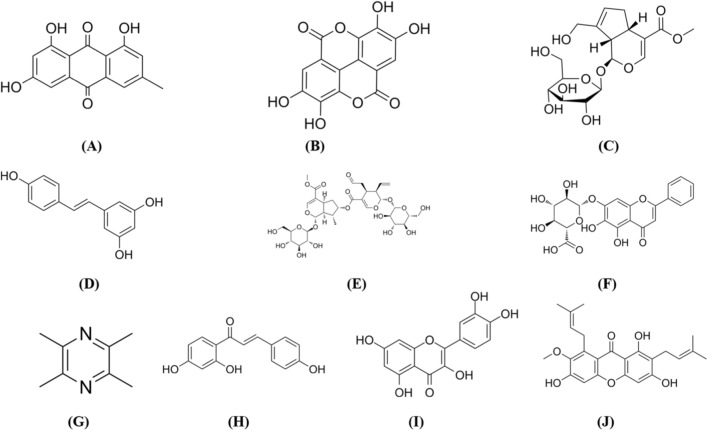
The structures of the metabolites mentioned in the article. **(A)** Emodin; **(B)** Ellagic Acid; **(C)** Geniposide; **(D)** Resveratrol; **(E)** Cantleyoside; **(F)** Baicalin; **(G)** Ligustrazine **(H)** Isoliquiritigenin; (**(I)** Quercetin; **(J)** α-Mangostin.

Emodin (Figure 6A), a natural anthraquinone derivative isolated from the roots and rhizomes of *Rheum palmatum* L. and other medicinal plants, exerts precise molecular interactions and inhibitory activity owing to its tricyclic diketone structure. This structure serves as a cap group for HDAC, enabling an interaction with amino acid residues at the HDAC active site (Zou et al., 2020). Under hypoxic conditions, emodin exhibits dose-dependent effects on IL-1β/LPS-co-stimulated primary RA synoviocytes. At concentrations of 0.1–10 μM, it dose-dependently inhibits cell proliferation, reduces the secretion of pro-inflammatory cytokines (TNF-α, IL-6, IL-8), inflammatory mediator (PGE_2_), matrix metalloproteinases (MMP-1, MMP-13), and angiogenic factor VEGF, and downregulates the mRNA expression of COX-2, VEGF, HIF-1α, MMP-1, and MMP-13. Notably, emodin specifically inhibits HDAC1 expression without affecting HDAC2 levels, NF-κB DNA-binding activity, or the phosphorylation of ERK and p38 MAPK. Collectively, these findings indicate that emodin exerts anti-RA effects by inhibiting synoviocyte proliferation, alleviating inflammatory responses and angiogenesis, and targeting the HDAC1-mediated signaling pathway (Ha et al., 2011). Future studies should clarify the specific mechanism of HDAC1 using gene manipulation approaches, such as HDAC1-specific siRNA knockdown or CRISPR-Cas9 knockout.

Ellagic acid (Figure 6B) is a natural polyphenol that is found in *Terminalia chebula* Retz. and other medicinal plants. Studies have demonstrated that polyphenols interact with HDACs through specific structural features validated by molecular docking. Phenolic hydroxyl groups form hydrogen bonds with amino acid residues in the HDAC active site, and aromatic rings establish hydrophobic interactions and π-π stacking in the hydrophobic pocket to enhance binding affinity (Uba and Zengin, 2023). *In vitro*, ellagic acid exerts therapeutic effects on TNF-α-induced MH7A cells, with 50 μM identified as the optimal dose. It dose-dependently inhibits cell proliferation, reduces IL-6 and IL-1β secretion, alleviates oxidative stress, promotes cell apoptosis, downregulates MTA1 and HDAC1 expression, and suppresses MTA1/HDAC1 complex formation. ChIP assays confirm that ellagic acid blocks HDAC1-mediated Nur77 deacetylation, leading to upregulated Nur77 expression. *In vivo*, ellagic acid significantly ameliorates CIA in male Wistar rats when administered orally at 25, 50, and 100 mg/kg/day for 21 days, with 50 mg/kg/day identified as the optimal dose. This dosage reduces arthritis index and paw swelling, attenuates synovial hyperplasia and inflammatory cell infiltration, and decreases TNF-α, IL-6, and IL-1β levels in both synovial tissue and serum. These studies have shown that ellagic acid can play an anti-RA role by targeting the MTA1/HDAC1-Nur77 signaling axis, inhibiting MTA1 to reduce HDAC1 levels and prevent Nur77 deacetylation, thereby enhancing Nur77 transcriptional activity to suppress synoviocyte hyperproliferation, inflammation, and oxidative stress while promoting cell apoptosis (Song et al., 2021).

Geniposide (Figure 6C) is an iridoid glycoside derived from the dried mature fruits of *Gardenia jasminoides* Ellis and other medicinal plants, known for its anti-inflammatory, anti-angiogenesis, and immune-regulating activities. Studies indicate that Geniposide acts on SphK1-activated HUVECs at concentrations of 25, 50, and 100 μM. Its dose-dependently inhibits proliferation, migration, and capillary-like tube formation, reduces S1P secretion, and downregulates SphK1, p-SphK1, S1PR1, PI3K, p-PI3K, Akt, p-Akt, HDAC3/6, and HIF-1α expression, while restoring ac-HIF-1α levels and inhibiting VEGF/VEGFR2 transcription. *In vivo*, Geniposide treats CIA in SD rats through intragastric administration at doses of 30, 60, and 120 mg/kg/day for 21 days, with 60 mg/kg/day identified as optimal. This treatment reduces arthritis index, paw swelling, and joint surface temperature, improves synovial pathology, and diminishes blood flow signals in the knee joint. It decreases Vimentin, NG2, and CD31 expression and lowers S1P and VEGF levels in serum and synovial tissue. High-dose Geniposide exhibits efficacy comparable to methotrexate in this model. Mechanistically, Geniposide negatively regulates SphK1 activation and downregulates the S1P-S1PR1-PI3K- Akt pathway, inhibiting HDAC3/6 activation. It promotes HIF-1α acetylation and degradation by increasing ac-HIF-1α levels, thereby blocking VEGF/VEGFR2 transcription. This action suppresses synovial angiogenesis in RA, affirming Geniposide’s anti-RA effects through inhibition of vascular angiogenesis, regulation of immune homeostasis, and targeting of the S1P-S1PR1-PI3K-Akt-HDAC3/6-HIF-1α-VEGF/VEGFR2 signaling axis (Li, 2025).

### 4.2 TCM extracts

*Ephedra sinica* polysaccharide are crude polysaccharide fractions derived from the dried herbaceous stems of *E. sinica* Stapf. Composed of glucose, arabinose, and galacturonic acid. Studies have shown that it acts on mouse RA-FLS at concentrations of 5–20 μg/mL *in vitro*, dose-dependently inhibiting proliferation and migration while suppressing IL-1β/TNF-α-induced inflammatory responses. *In vivo*, it effectively treats CIA in C57BL/6J mice through oral administration at doses of 200 and 400 mg/kg/day for 21 days, with 400 mg/kg/day identified as the optimal dose. This treatment significantly reduces joint swelling and arthritis severity, decreases serum IL-1β and IL-6 levels, and alleviates synovial hyperplasia, inflammatory cell infiltration, cartilage destruction, and bone erosion, showed comparable reductions in arthritis scores to methotrexate in this preclinical model. It also restores intestinal barrier integrity by upregulating tight junction proteins (e.g., occludin and ZO-1) and modulating gut microbiota composition. Mechanistically, *E. sinica* polysaccharide modulates gut microbiota dysbiosis in CIA mice by increasing populations of anti-inflammatory and SCFA-producing bacteria such as *Dubosiella*, *Bifidobacterium*, and *Clostridium*, while decreasing pro-inflammatory bacteria like *Alistipes* and *Enterococcus*. It regulates serum metabolites by increasing L-tyrosine and butyrate and decreasing N-palmitoyl taurine. Additionally, it acts through the TLR4/HDAC/NF-κB signaling pathway by downregulating TLR4, MyD88, and TRAF6 expression, inhibiting NF-κB p65 phosphorylation, and blocking HDAC1 and HDAC2 activity. These studies confirm its anti-RA effects by regulating gut microecological balance, modulating microbial metabolites, repairing intestinal barrier function, and inhibiting the TLR4/HDAC/NF-κB inflammatory signaling pathway (Ma et al., 2024).

### 4.3 TCM formulas

In a broader therapeutic context, TCM formulas such as Guizhi-Shaoyao-Zhimu Decoction offer comprehensive strategies for combating inflammation in RA. It exhibits multiple biological activities such as anti-inflammation, immune regulation, inhibition of osteoclast differentiation, and modulation of synoviocyte function. Studies suggest that it acts on IL-1β-induced human HFLS-RA *in vitro* at concentrations of 5.12 × 10^−5^, 2.56 × 10^−4^, and 1.28 × 10^−3^ μg/mL, dose-dependently downregulating the abnormally elevated proteins HDAC1, HSP90AA1, NFKB2, IKBKB, and TNF-α in RA synovial cells. *In vivo*, the decoction treats AIA in Lewis rats through intragastric administration at 4.65, 9.3, and 18.6 g/kg/day for 21 days, with 18.6 g/kg/day identified as the optimal dose. This treatment significantly alleviates joint redness and swelling, reduces arthritis score, incidence, and percentage of arthritic limbs, delays arthritis onset, and mitigates synovial hyperplasia, inflammatory cell infiltration, cartilage degradation, and bone destruction, demonstrating therapeutic efficacy comparable to methotrexate in this model. Mechanistically, it reverses the inflammation-immune system imbalance in RA by regulating the HDAC1-HSP90AA1-NFKB2-IKBKB-TNF-α signaling axis. This axis connects multiple RA-related pathways, including T/B cell receptor, Toll-like receptor, NF-κB, TNF signaling, and osteoclast differentiation. Long-term clinical trials confirm its high cure rate and safety, underscoring its significant clinical value (Guo et al., 2016).

Fengshining exhibits a variety of biological activities, such as anti-inflammation, gut microbiota regulation, intestinal barrier repair, and inhibition of the HDAC/NF-κB signaling pathway. *In vitro* studies show that Fengshining Decoction -containing serum inhibits the proliferation and migration of RA-FLS, promotes apoptosis, and alleviates TNF-α-induced abnormal reorganization of the F-actin cytoskeleton. *In vivo*, the decoction treats CIA in mice via intragastric administration at doses of 20, 40, and 80 g/kg/day over 21 days, with 40 g/kg/day identified as the optimal dose. This treatment significantly reduces joint swelling and deformity, lowers serum IL-1β and IL-6 levels, and improves joint pathology by mitigating synovial hyperplasia, inflammatory cell infiltration, and bone/cartilage destruction. It restores intestinal barrier function by upregulating tight junction proteins ZO-1, Occludin, and mucin Mucin 2, increasing goblet cell numbers, and reducing intestinal mucosal erosion. Mechanistically, Fengshining Decoction modulates gut microbiota dysbiosis in CIA mice by increasing the abundance of SCFA-producing bacteria such as *Butyrivibrio*, *Faecalicatena*, and *Lacrimispora*, which enhances butyrate production. As an HDAC inhibitor, butyrate inhibits HDAC1/2 activity, blocks NF-κB p65 phosphorylation and nuclear translocation, and suppresses downstream inflammatory signaling cascades. Additionally, Fecal microbiota transplantation and exogenous butyrate supplementation replicate Fengshining Decoction’s effects, validating the gut microbiota-metabolite-HDAC/NF-κB axis (Wen et al., 2025).

Despite their different component, Fengshining Decoction and *E. sinica* polysaccharide work similarly in treating RA. They target HDAC1/2 to rebalance epigenetics, modulate NF-κB (Figure 3), and reduce cytokines such as TNF-α, IL-1β, and IL-6, along with MMPs. They act on the gut microbiota–metabolite–HDAC axis. They improve gut flora by increasing SCFA-producing bacteria and reducing harmful pathogens. This process boosts butyrate production. Butyrate strengthens the intestinal barrier by upregulating ZO-1, Occludin, and MUC2 and directly inhibits HDAC1/2. Fengshining Decoction blocks the HDAC/NF-κB pathway, while *E. sinica* polysaccharide targets the TLR4/MyD88-HDAC/NF-κB axis. Together, these actions reduce p65 phosphorylation and inhibit abnormal growth and movement of RA-FLS. This helps alleviate joint inflammation and tissue damage. The multi-target, multi-pathway approach of these formulas offers clear benefits over single-target treatments.

### 4.4 Translational readiness

TCM-derived HDACis show promising potential for RA therapy, grounded in a solid preclinical foundation. Evidence from *in vitro* studies involving RA FLS, HUVECs, and mouse FLS cell lines, as well as *in vivo* models like CIA and AIA, demonstrates their ability to target pro-inflammatory HDAC isoforms (HDAC1, 3, and 6). These inhibitors effectively hinder downstream pathways such as HDAC/NF-κB, S1P-PI3K-Akt-HIF-1α, and TLR4/MyD88, reduce pro-inflammatory cytokines (IL-6 and TNF-α) and VEGF, suppress synovial hyperplasia and joint damage, and modulate gut microbiota and intestinal barrier function. Notably, emodin has progressed to *ex vivo* human validation using RA patient-derived primary synoviocytes, increasing its relevance to human pathology. However, clinical translation faces significant challenges. Key obstacles include the lack of Phase I-IV clinical trials to confirm human safety, tolerability, and pharmacokinetic/pharmacodynamic profiles, alongside difficulties in achieving HDAC isoform specificity and concerns about off-target effects. Additionally, standardizing TCM formulations for consistent active metabolite quantification is difficult due to variability in herb sourcing and processing, and human-derived evidence is limited. Addressing these gaps requires early-phase clinical trials, expanded human study validation with RA patient-derived tissues, and advanced formulation strategies to enhance bioavailability and efficacy, paving the way for meaningful clinical applications in RA treatment.

## 5 Activators of anti-inflammatory HDAC isoforms

Exploration into activators of anti-inflammatory HDAC isoforms reveals a profound impact on epigenetic modulation by TCM. TCM encompasses a variety of chemical metabolites that interact with SIRTs, a family of NAD^+^ dependent protein deacetylases, profoundly influencing biological systems. These metabolites display potential in modulating inflammatory responses, oxidative stress, and metabolic pathways by either activating or inhibiting different SIRT isoforms. SIRT1 activators exert their biological effects by binding to an allosteric site on SIRT1, which is located in the two helices of the N-terminal domain and distinct from the catalytic domain. This binding induces a conformational change, bringing the N-terminal domain closer to the catalytic domain, thereby promoting the interaction between the SIRT1 substrate polypeptide and the catalytic domain and ultimately enhancing the catalytic activity of SIRT1 (An et al., 2020). Through these interactions, TCM metabolites can promote anti-inflammatory and antioxidative effects, offering pathways for mitigating chronic conditions and enhancing metabolic health (Table 3).

**TABLE 3:** The anti-RA effects and mechanisms of TCM activators.

Name	Compound class	Latin binomial	Experimental type	Model	Dosages tested (Route of administration)	Key outcomes	Safety signals	References
Resveratrol	polyphenol	-	*in vitro*	Human (THP-1-derived macrophages), n = 3 replicate experiments; induced by LPS (100 ng/mL) + ATP (5 mM) or ACPA (purified from RA patient serum)	7.5, 15, 30 μM(pretreated for 6 h; stimulated for 1–4 h)	NLRP3 inflammasome activation↓; caspase-1 p20; IL-1β p17↓; SIRT1↑(mediates NLRP3 deacetylation); ITGB α5β1 competitive binding↑; ATP release↓; pyroptosis↓	No obvious cytotoxicity at ≤30 μM; Fewer side effects reported	Cai et al. (2025)
			*in vivo*	DBA/1J mice, n = 3–6 per group; induced by chicken type II collagen + CFA/IFA (CIA) or citrullinated type II collagen + CFA/IFA (Cit-CIA)	2.5 mg/kg (intraperitoneal injection, once daily from booster immunization for 21 days)	Arthritis index; paw swelling↓; synovial inflammation; bone destruction↓; serum TNF-α; IL-6; IL-1β↓; synovial NLRP3; caspase-1 p20↓; SIRT1↑	Fewer side effects reported	Cai et al. (2025)
			*in vitro*	Human (RA FLS), n = 3 replicate experiments, no induction; isolated from RA patient synovial tissues	1, 3, 10 µg/mL (incubated for 48 h)	SIRT1 (protein + mRNA) ↑; RA FLS invasiveness↓; MMP1; MMP13 (protein + mRNA) ↓; cell viability: no change	No obvious cytotoxicity	Hao et al. (2017)
			*in vivo*	SD rats, n = 10 per group, induced by subcutaneous injection of type II collagen + complete Freund’s adjuvant emulsion (primary immunization on day 0, booster on day 7)	2.5, 10 mg/kg (intraperitoneal injection, once daily for 42 consecutive days)	Arthritis index↓; joint swelling; bone destruction↓; synovial SIRT1 (protein + mRNA)↑; MMP1; MMP13 (protein + mRNA)↓(dose-dependent)	No significant side effects reported	Hao et al. (2017)
			*in vitro*	Human (RASFs), n = 5 independent experiments, induced by bradykinin (BK, 1 μM) via B2R receptor stimulation	0.2, 2, 20 μM(pretreated for 1 h; stimulated for 6 h)	SIRT1↑; COX-2 (protein + mRNA); PGE_2_↓; AP-1 (c-Jun/c-Fos); NF-κB p65 (phosphorylation + acetylation)↓; AP-1/NF-κB binding to COX-2 promoter↓; PKCι; MAPKs (ERK1/2; p38; JNK1/2) signaling↓	No obvious cytotoxicity	Yang et al. (2017)
​	​	​	*in vitro*	Human (MH7A), n = 3–8 independent experiments; direct treatment with resveratrol	1, 10, 50, 100, 200 μM(treated for 24–72 h; 100 μM for main experiments)	Cell viability↓(dose- and time-dependent); apoptosis↑(TUNEL-positive cells; H2A.X phosphorylation↑); mitochondrial membrane potential disruption; cytochrome c release↑; caspase-3/-9 activation↑(caspase-8: no change); SIRT1 mRNA↑; Bcl-XL mRNA↓; effects blocked by Sirtinol	No obvious cytotoxicity-related details provided	Nakayama et al. (2010)
			*in vitro*	1. Canine (primary bone-derived cells), n = 3 independent experiments, induced by RANKL (10 nM) in high-density/monolayer cultures; 2. Mouse (preosteoblastic cell line MC3T3-E1), n = 3 independent experiments, induced by RANKL (10 nM) in differentiation medium	5 μM(pretreated for 4 h; co-treated with RANKL for 3–14 days)	Osteoclastogenesis↓(TRAP + multinucleated cells↓); NF-κB activation↓(IKK activity; IκB phosphorylation/degradation; p65 acetylation/nuclear translocation↓); SIRT1↑; p300↓; SIRT1-p300 complex↑; Cbfa-1↑; bone matrix (collagen type I; osteocalcin)↑; RANKL-induced osteogenic inhibition reversed	No cytotoxicity reported at tested concentration	Shakibaei et al. (2011)
			*in vitro*	Rat (primary fibroblast-like synoviocytes, FLS), n = 3 independent experiments, induced by H_2_O_2_ (5 μM) stimulation for 12 h	100 μM(pretreated for 24 h; combined with SIRT1 inhibitor sirtinol or si-Nrf2 for mechanism validation)	ROS production↓; FLS proliferation↓; apoptosis↑; SIRT1/Nrf2/HO-1/NQO1↑; NF-κB p65 (phosphorylation + acetylation)↓; miR-29a-3p; miR-23a-3p↑; Keap1↓(targeted by miR-29a-3p); Cul3↓(targeted by miR-23a-3p)	No obvious cytotoxicity at tested concentration	Wang et al. (2019)
			*in vivo*	SD rats, n = 6 per group, induced by intradermal injection of complete Freund’s adjuvant (CFA) (model established on day 0)	10 mg/kg (oral gavage, once daily from day 12 to day 24 after model establishment)	Arthritis index; paw swelling↓; synovial hyperplasia; inflammatory infiltration; cartilage degradation↓; serum MDA↓; SOD; GSH-Px↑; synovial SIRT1; Nrf2; HO-1; NQO1↑; Ki-67^+^ cells↓(inhibits synoviocyte proliferation)	No significant side effects reported	Wang et al. (2019)
Cantleyoside	Iridoid glycoside	-	*in vitro*	Human (HFLS-RA), n = 3 independent experiments, induced by LPS (1 μg/mL) stimulation for 24 h (pretreated with CA for 4 h)	10, 20, 40 μM(main experiments); 1, 500 μM(cell viability assay)	HFLS-RA proliferation; migration↓; NO; TNF-α; IL-1β/6; MCP-1; MMP-1/3/9↓; mitochondrial dysfunction (MMP↓; ROS/Ca^2+^↑; MPTP opening↑; OCR/ECAR↓; ATP↓); apoptosis↑(TUNEL/Hoechst-positive cells↑; Bax/Bcl-2 ratio↑); AMPK/p-AMPK; SIRT1↑; IκBα degradation; NF-κB p65 phosphorylation/nuclear translocation↓	≤100 μM no obvious cytotoxicity; 500 μM exhibits cytotoxicity	Bai et al. (2022)
Baicalin	Flavonoid	-	*in vitro*	Human (HFLS-RA), n = 3 independent experiments, no induction; direct treatment with baicalin	10, 20, 30 μM(incubated for 24 h); 40, 50, 60 μM(incubated for 48 h)	HFLS-RA proliferation↓; TNF-α; IL-1β secretion↓; NF-κB p65 (protein expression + Ser536 phosphorylation + Lys310 acetylation)↓; SIRT1↑(promotes p65 deacetylation)	No obvious cytotoxicity at tested concentrations	Guo et al. (2024)
			*in vivo*	Wistar rats, n = 6 per group, induced by bovine type II collagen + complete Freund’s adjuvant (primary immunization on day 0, booster on day 7 with incomplete Freund’s adjuvant)	50, 100, 200 mg/kg (intraperitoneal injection, once daily from day 16 to day 46 after primary immunization, total 30 days)	Arthritis index; paw swelling↓; synovial inflammation; hyperplasia; pannus formation↓; cartilage/bone destruction↓; serum TNF-α; IL-1β↓; synovial NF-κB p65 (protein + phosphorylation + acetylation)↓; SIRT1↑(dose-dependent)	No significant side effects reported	Guo et al. (2024)
Ligustrazine	Alkaloid	-	*in vivo*	SD rats, n = 8 per group, induced by subcutaneous injection of FCA in left hind paw on day 0	10, 20 mg/kg (oral gavage, once daily from day 21 to day 28); Diclofenac sodium 5 mg/kg	Hind paw swelling↓; joint pathology improvement (articular cartilage lesion; inflammatory infiltration; synovial hyperplasia↓); serum IL-6; IL-1β; TNF-α↓; SOD activity↑; MDA↓; synovial SIRT1↑; p-NF-κB p65; p-IκBα↓; Nrf2 nuclear translocation↑; HO-1↑	No significant side effects reported	Li Y. Z. et al. (2018)
Isoliquiritigenin	Flavonoid	-	*in vitro*	Human (chondrocyte cell line, C20A4), n ≥ 3 independent experiments, induced by IL-1β (20 ng/mL) + TNF-α (20 ng/mL) stimulation for 48 h (pretreated with ISL for 1 h)	3, 5, 10 μM(pretreated for 1 h; combined with Nrf2 inhibitor Brusatol or HO-1 inhibitor Znpp for mechanism validation)	viability↑; ROS↓; apoptosis↓(Bax↓; c-caspase-3↓; Bcl-2↑); MMP13; iNOS↓; Nrf2; HO-1; PGC-1α; SIRT1↑; effects reversed by Brusatol/Znpp	No obvious cytotoxicity at tested concentrations	Hung et al. (2024)
​	​	​	*in vivo*	SD rats, n ≥ 3 per group, induced by adjuvant-induced arthritis (AIA: CFA + IFA + mBSA injection)	10 mg/kg (intra-articular injection, once daily for 3 consecutive days)	Arthritis index↓; behavior score improved; paw thickness↓; cartilage damage↓; joint cartilage Nrf2/Bcl-2↑; iNOS/Bax↓; skeletal muscle oxidative stress↓(MDA; nitrotyrosine↓); serum MMP13↓; Nrf2/HO-1/SIRT1↑ in muscle tissue	No significant side effects reported	Hung et al. (2024)
Quercetin	Flavonoid	-	*in vivo*	C57BL/6 mice (7-week-old), n = 8 per group, induced by collagen-induced arthritis (CIA: chicken type II collagen + complete/incomplete Freund’s adjuvant, primary immunization on day 0, booster on day 21)	50 mg/kg (oral gavage), 100 mg/kg (oral gavage)(once daily from day 22 to day 57, total 5 weeks)	Arthritis index; ankle thickness↓; synovial inflammation; hyperplasia; pannus formation; bone/cartilage destruction↓; serum/joint IL-6; TNF-α; IL-1β; IL-8; IL-13; IL-17↓; anti-CII IgG1/IgG2a↓; mitochondrial biogenesis improved (mtDNA; CO1/CO2/CO3/ATP6/ATP8↑; SOD/CAT/GSH-Px↑; MDA↓); SIRT1/PGC-1α/NRF1/TFAM↑; HMGB1/TLR4/P-p38/P-ERK1/2/P-NF-κB p65↓	No obvious side effects reported	Shen et al. (2021)
α-Mangostin	Isoprenyl-substituted xanthone	-	*in vivo*	SD rats, n = 6 per group; induced by bovine type II collagen + complete Freund’s adjuvant (primary immunization on day 0, booster immunization on day 7)	40 mg/kg (oral gavage, once daily; early treatment: day 0–20; late treatment: day 21–40)	Arthritis symptoms↓; joint pathology improved (synovial hyperplasia; inflammatory infiltration; pannus formation; cartilage erosion↓); M1 macrophage polarization (CD11b^+^CD86^+^) ↓; Th1 cell differentiation (CD4^+^IFN-γ^+^) ↓; serum AChE activity↓; NAD^+^ level↑; α7nAChR and SIRT1↑; NF-κB p65 (phosphorylation + acetylation) ↓	No significant side effects reported	Chen W. et al. (2022)
			*in vitro*	1. Mouse macrophage cell line (RAW264.7), n = 3 replicate experiments, induced by LPS (2 nM); 2. Rat peritoneal macrophages, isolated from AIA rats	5 μg/mL (*in vitro* experiments); α7nAChR inhibitor MLA (5 nM) used for mechanism validation	LPS-induced inflammation↓; α7nAChR and SIRT1↑; NF-κB p65 phosphorylation↓; MLA reversed the upregulatory effect of α-Mangostin on α7nAChR/SIRT1; α7nAChR and SIRT1↑; p65 acetylation↓in rat peritoneal macrophages	No obvious cytotoxicity at tested concentration	Chen W. et al. (2022)
*Litsea salicifolia* Roxb. bark ethanolic extract	flavonoids, alkaloids, sesquiterpenesetc.	*Litsea salicifolia* (Roxb. ex Nees) Hook.f.	*in vitro*	Mouse macrophage cell line (RAW 264.7), n = 3 replicate experiments, induced by LPS (1 μg/mL) stimulation	2.5, 5, 10 μg/mL (pretreated for 2 h; LPS stimulation for 22 h); 5, 10 μg/mL (for cytokine/protein detection)	ROS; NO; PGE2↓; IL-1β; IL-6; TNF-α↓; TLR4; NF-κB p65 (phosphorylation); COX-2; iNOS↓; SIRT1; Nrf2; HO-1↑; NF-κB p65 nuclear translocation↓	No cytotoxicity at ≤100 μg/mL	Puppala et al. (2023)
​	​	​	*in vivo*	Wistar rats, n = 6 per group, induced by intradermal injection of Freund’s complete adjuvant (CFA, 100 μL) on day 1	100, 200 mg/kg (oral gavage, once daily from day 1 to day 28); Diclofenac sodium 15 mg/kg	Arthritis index; paw volume↓; body weight recovery↑; synovial hyperplasia; inflammatory infiltration; bone erosion↓; MDA; NO↓; GSH↑; IL-1β; IL-6; TNF-α; IFN-γ↓; IL-10↑; TLR4; NF-κB p65 (phosphorylation); COX-2; iNOS↓; SIRT1; Nrf2; HO-1↑; spleen index↓; thymus index↑; IL-6; TGF-β; MMP-2; collagen II mRNA↓	No toxicity at 2000 mg/kg (acute toxicity test)	Puppala et al. (2023)
Qufeng epimedium decoction	-	*Epimedium brevicornu* Maxim *Saposhnikovia divaricata* (Turcz.) Schischk *Spatholobus suberectus* Dunn *Erythrina variegata* L. var. *orientalis* (L.) Merr *Vespa velutina* Lepeletier *Astragalus mongholicus* Bunge *Wolfiporia extensa* (Peck) Ginns *Paeonia lactiflora* Pall *Cyperus rotundus* L *Rehmannia glutinosa* (Gaertn.) DC. *Psoralea corylifolia* L Campsis grandiflora (Thunb.) K.Schum Liquidambar formosana Hance Glycyrrhiza uralensis Fisch	*in vitro*	Rat (FLSs), isolated from RA model rats, n = 3 replicate experiments, no additional induction; treated with decoction-related interventions	Isolated FLSs from RA rats (treated with decoction *in vivo*); transfected with sh-CYLD/sh-NC for mechanism validation	FLS pyroptosis↓(Caspase-1^+^PI^+^ cells; GSDMD; NLRP3↓); SIRT1 protein stability↑(ubiquitination↓); CYLD↑(mediates SIRT1 deubiquitination); NF-κB p65 phosphorylation↓; IL-6; TNF-α; IL-1β; IL-18↓	No obvious toxicity reported	Wu et al. (2025)
			*in vivo*	SD rats, n = 5 per group, induced by intradermal injection of CFA, 0.1 mL in left hind footpad on day 0	3.2 g/kg (oral gavage, once daily from day 12 after model establishment)	Arthritis index; paw swelling↓; synovial hyperplasia; inflammatory infiltration; cartilage/bone erosion↓; Ki67^+^ proliferating cells↓; IL-6; TNF-α↓; FLS pyroptosis↓(GSDMD; NLRP3↓); SIRT1↑(deubiquitinated by CYLD); CYLD↑; NF-κB/GSDMD signaling pathway↓	No significant side effects reported	Wu et al. (2025)
Bushen Jiedu Tongluo Formula	-	*Curculigo orchioides* Gaertn *Epimedium brevicornu* Maxim *Dipsacus asper* Wall. ex DC. *Sarcandra glabra* (Thunb.) Nakai *Sinomenium acutum* (Thunb.) Rehder and E.H.Wilson *Trachelospermum jasminoides* (Lindl.) Lem *Paeonia veitchii* Lynch *Curcuma zedoaria* (Christm.) Roscoe *Paeonia suffruticosa* Andrews *Curcuma wenyujin* Y.H.Chen and C.Ling *Buthus martensii* Karsch *Zaocys dhumnades* (Cantor) *Glycyrrhiza uralensis* Fisch	*in vivo*	SD rats, n = 10 per group (High dose group n = 9), induced by collagen-induced arthritis (CIA: bovine type II collagen + incomplete Freund’s adjuvant) + HMGB1 (100 μg/kg, intra-articular injection, twice a week for 3 weeks)	Low: 0.98 g/mL, Middle: 1.95 g/mL, High: 3.9 g/mL (oral gavage, once daily for 21 days); SRT1720 0.1 g/kg	Arthritis index; paw thickness↓; synovial inflammation; pannus formation↓; synovial microvessel density (MVD); CD31^+^ expression↓; SIRT1 (protein + mRNA)↑; HMGB1; NF-κB (p65); HIF-1α; VEGF (protein + mRNA)↓; HMGB1; HIF-1α acetylation level↓; dose-dependent effect	No obvious toxicity; 1 rat in High dose group died of gavage operation error	Chen (2021)
			*in vitro*	Human (HUVECs), n = 3 replicate experiments, induced by HMGB1 (100 ng/mL, pretreatment for 24 h)	Low: 0.25 mg/mL, Middle: 0.5 mg/mL, High: 1 mg/mL (*in vitro* culture); SRT1720 5 μM	Cell proliferation; migration; capillary-like tube formation↓; SIRT1 protein↑; HMGB1; NF-κB (p65); HIF-1α; VEGF protein↓; dose-dependent effect	No cytotoxicity at tested concentrations	Chen (2021)
Wutou Decoction	-	*Ephedra sinica* Stapf *Aconitum carmichaelii* Debeaux *Glycyrrhiza uralensis* Fisch *Paeonia lactiflora* Pall *Astragalus mongholicus* Bunge	*in vivo*	SD rats, n = 10 per group, induced by collagen-induced arthritis (CIA: bovine type II collagen + complete/incomplete Freund’s adjuvant, primary immunization + booster after 7 days)	Low: 3.8 g/kg, High: 7.6 g/kg (oral gavage, once daily from day 9 to day 51); Methotrexate 2 mg/kg (oral gavage, once weekly)	Arthritis index (AI; ankle thickness↓; synovial inflammation; synovial hyperplasia; pannus formation; bone/cartilage destruction↓; M1 macrophage polarization (F4/80^+^iNOS^+^ cells)↓; TNF-α; IL-1β; IL-6; IL-12↓; HMGB1 acetylation↓; nuclear translocation↑; cytoplasmic HMGB1↓; TLR4; RAGE; NF-κB p65 nuclear translocation↓; SIRT1 (protein + deacetylase activity) ↑; NAD+/NADH ratio↑	No obvious hepatorenal toxicity	Shen et al. (2025)
			*in vitro*	Mouse (RAW264.7), n = 3 replicate experiments, induced by LPS (1 μg/mL) stimulation for 12 h (pretreated with WTD-containing serum for 12 h)	containing serum: 10%, 15%; SIRT1 inhibitor EX527 (16 μM) for mechanism validation	LPS-induced M1 macrophage invasion↓; iNOS; COX2↓; TNF-α; IL-1β; IL-6; IL-12↓; HMGB1 acetylation↓; cytoplasmic HMGB1↓; nuclear HMGB1↑; NF-κB p65 nuclear translocation↓; SIRT1↑; deacetylase activity↑; EX527 reverses WTD’s effects	No obvious cytotoxicity at tested concentrations	Shen et al. (2025)

↑ = Increase/Promote/Upregulate; ↓ = Decrease/Inhibit/Downregulate; AChE, acetylcholinesterase; AIA, Adjuvant-Induced Arthritis; α7nAChR, α7 Nicotinic Acetylcholine Receptor; AMPK, AMP-Activated Protein Kinase; AP-1, Activator Protein-1; ATP, adenosine triphosphate; Bax, Bcl-2-Associated X Protein; Bcl-2, B-Cell Lymphoma-2; Bcl-XL, B-Cell Lymphoma-Extra Large; BK, bradykinin; B2R, Bradykinin B2 receptor; CA, cantleyoside; Cbfa-1, Core Binding Factor Alpha 1; CAT, catalase; Caspase, Cysteine Aspartate-Specific Protease; c-caspase-3, Cleaved Caspase-3; C20A4, Human Chondrocyte Cell Line C20A4; CD11b^+^CD86^+^, Cluster of Differentiation 11b^+^CD86^+^ (M1 Macrophage Marker); CD4^+^IFN-γ^+^, Cluster of Differentiation 4^+^Interferon-γ^+^ (Th1 Cell Marker); CD31, Platelet Endothelial Cell Adhesion Molecule-1; CFA, Complete Freund’s Adjuvant; Cit-CIA, Citrullinated Type II, Collagen-Induced Arthritis; CIA, Collagen-Induced Arthritis; COX-2, Cyclooxygenase-2; CYLD, cylindromatosis tumor suppressor; Cul3, Cullin 3; c-Fos, c-Fos Proto-Oncogene; c-Jun, c-Jun Proto-Oncogene; deubiquitination, Deubiquitination; DBA/1J mice, DBA/1J Mouse Strain; DS, diclofenac sodium; ECAR, extracellular acidification rate; ERK1/2, Extracellular-Signal-Regulated Kinase 1/2; EX527, SIRT1 Inhibitor EX527; FCA, Freund’s Complete Adjuvant; FLSs, Fibroblast-Like Synoviocytes; GSDMD, Gasdermin D; GSH, glutathione; GSH-Px, Glutathione Peroxidase; HO-1, Heme Oxygenase-1; HMGB1, High Mobility Group Box 1; HIF-1α, Hypoxia-Inducible Factor-1α; Hoechst, Hoechst Fluorescent Dye; HFLS-RA, Human Fibroblast-Like Synoviocyte from Rheumatoid Arthritis; H_2_O_2_, hydrogen peroxide; H2A.X, Histone H2A.X; IFA, Freund’s Incomplete Adjuvant; IκB, Inhibitor of NF-κB; IKK, IκB kinase; IL, interleukin; IL-1β, Interleukin-1, beta; IL-6, Interleukin-6; IL-8, Interleukin-8; IL-10, Interleukin-10; IL-12, Interleukin-12; IL-13, Interleukin-13; IL-17, Interleukin-17; IL-18, Interleukin-18; iNOS, inducible nitric oxide synthase; ISL, isoliquiritigenin; ITGB α5β1, Integrin α5β1; JNK1/2, c-Jun N-Terminal Kinase 1/2; Keap1, Kelch-Like ECH-Associated Protein 1; Ki-67, Ki-67 Proliferation Marker; LPS, lipopolysaccharide; Litsea salicifolia Roxb. bark ethanolic extract, Litsea salicifolia Roxb. bark ethanolic extract; M1 macrophage, Pro-Inflammatory Macrophage; MDA, malondialdehyde; MC3T3-E1, Mouse Preosteoblastic Cell Line MC3T3-E1; MCP-1, Monocyte Chemoattractant Protein-1; MMP, matrix metalloproteinase; MMP-1, Matrix Metalloproteinase-1; MMP-3, Matrix Metalloproteinase-3; MMP-9, Matrix Metalloproteinase-9; MMP-13, Matrix Metalloproteinase-13; MLA, methyllycaconitine; mtDNA, Mitochondrial DNA; MPTP, mitochondrial permeability transition pore; MVD, microvessel density; mBSA, methyl bovine serum albumin; MyD88, Myeloid Differentiation Primary Response 88; NAD^+^, nicotinamide adenine dinucleotide; NAD+/NADH, Nicotinamide Adenine Dinucleotide/Nicotinamide Adenine Dinucleotide Hydrogen; NF-κB, Nuclear Factor Kappa-B; NRF1, Nuclear Respiratory Factor 1; Nrf2, Nuclear Factor-Erythroid 2-Related Factor 2; NLRP3, NOD-Like Receptor Protein 3; NO, nitric oxide; nitrotyrosine, Nitrotyrosine; NQO1, NAD(P)H Quinone Dehydrogenase 1; Occludin, Occludin; OCR, oxygen consumption rate; osteocalcin, Osteocalcin; PGE_2_, Prostaglandin E_2_; PGC-1α, Peroxisome Proliferator-Activated Receptor Gamma Coactivator 1α; PKCι, Protein Kinase Cι; PI, propidium iodide; PI3K, Phosphoinositide 3-Kinase; p-AMPK, Phosphorylated AMP-Activated Protein Kinase; p-IκBα, Phosphorylated Inhibitor of NF-κB, alpha; p-NF-κB p65, Phosphorylated Nuclear Factor Kappa-B p65 Subunit; p-PI3K, Phosphorylated Phosphoinositide 3-Kinase; p38, p38 Mitogen-Activated Protein Kinase; p65, Nuclear Factor Kappa-B p65 Subunit; RAGE, receptor for advanced glycation end products; Ras, Rat Sarcoma Viral Oncogene Homolog; RANKL, Receptor Activator of Nuclear Factor Kappa-B ligand; RASFs, Rheumatoid Arthritis Synovial Fibroblasts; RA, rheumatoid arthritis; ROS, reactive oxygen species; SIRT1, Sirtuin 1; SRT1720, SIRT1 Activator SRT1720; sirtinol, SIRT1 inhibitor sirtinol; sh-CYLD, Short Hairpin RNA, Targeting CYLD; sh-NC, Negative Control Short Hairpin RNA; SphK1, Sphingosine Kinase 1; SOD, superoxide dismutase; Ser536, Serine 536 (p65 Phosphorylation Site); Lys310, Lysine 310 (p65 Acetylation Site); TFAM, Mitochondrial Transcription Factor A; TGF-β, Transforming Growth Factor-β; TLR4, Toll-Like Receptor 4; TNF-α, Tumor Necrosis Factor-α; TRAF6, Tumor Necrosis Factor Receptor-Associated Factor 6; TRAP, Tartrate-Resistant Acid Phosphatase; TUNEL, Terminal Deoxynucleotidyl Transferase-Mediated dUTP, Nick-End Labeling; ubiquitylation, Ubiquitylation; VEGF, vascular endothelial growth factor; Znpp, Zinc Protoporphyrin; Brusatol, Nrf2 Inhibitor Brusatol; CO1/CO2/CO3/ATP6/ATP8, Mitochondrial Genes (Cytochrome c Oxidase Subunit 1/2/3, ATP, Synthase Subunit 6/8); Q-L/Q-H, Quercetin Low/High Dose; Ca^2+^, Calcium Ion; cytochrome c, Cytochrome c; pyroptosis, Pyroptosis; miR, microRNA; miR-29a-3p, microRNA-29a-3p; miR-23a-3p, microRNA-23a-3p.

### 5.1 TCM bioactive compounds

Resveratrol (Figure 6D) is a natural polyphenolic metabolite that is widely found in *Polygonum cuspidatum* Sieb. et Zucc and other medicinal plants, and it exhibits multiple biological activities, including anti-inflammatory, antioxidant, pro-apoptotic, and osteoclastogenesis-inhibiting effects. It is endowed with a unique chemical structure characterized by two benzene rings linked by a trans-stilbene backbone and three hydroxyl groups. The trans-stilbene backbone’s planar low-tension structure fits SIRT1’s hydrophobic cleft, stabilizing its active conformation, while the 3,5,4′-hydroxyl groups form hydrogen bonds to conserved amino acids in SIRT1’s catalytic domain, boosting binding affinity. Together, these mediate SIRT1 activation and weak SIRT2 inhibition, with trans-planarity critical for SIRT modulation (Melita et al., 2018). It activates SIRT1 to mediate the deacetylation of NF-κB p65, inhibiting its nuclear translocation and transcriptional activity, thereby reducing the secretion of pro-inflammatory cytokines such as TNF-α and IL-6; meanwhile, it deacetylates the subunits of activator protein-1 including c-Jun/c-Fos to attenuate inflammatory signal transduction (Yang et al., 2017). Through the regulation of inflammatory cascades, Resveratrol’s role becomes distinctively crucial. Relying on SIRT1, it directly deacetylates the NLRP3 inflammasome, blocking its assembly and activation, which in turn reduces the cleavage of caspase-1 and the release of mature IL-1β, and inhibits the amplification of the inflammatory cascade (Cai et al., 2025). Through the SIRT1/NF-κB/miR-29a-3p/Keap1 and SIRT1/NF-κB/miR-23a-3p/Cul3 signaling axes, it stabilizes the nuclear localization of Nrf2 and activates the Nrf2/antioxidant response element pathway. This upregulates the expression of antioxidant enzymes such as HO-1 and NAD(P)H, reduces the accumulation of reactive oxygen species in RA-FLS, and alleviates oxidative stress-induced damage (Wang et al., 2019). It enhances SIRT1 activity to suppress the abnormal proliferation, migration, and invasion of RA-FLS, downregulates the expression of MMPs such as MMP1 and MMP13. This multifaceted cascade elucidates resveratrol’s therapeutic potential. At the same time, it induces RA-FLS apoptosis by downregulating Bcl-XL and activating the mitochondrial apoptotic pathway, thereby reducing synovial hyperplasia and invasion (Hao et al., 2017). It promotes the formation of a complex between SIRT1 and the osteogenic transcription factor Cbfa-1, enhancing the proliferation and mineralization of osteoblasts. Meanwhile, it inhibits RANKL-induced osteoclast differentiation, improving RA-related bone destruction (Nakayama et al., 2010). Collectively, resveratrol demonstrates a single-core, multi-pathway mechanism centered on SIRT1 activation.

Cantleyoside (Figure 6E), an iridoid glycoside isolated from the *Pterocephalus hookeri* (C. B. Clarke) Höeck and other medicinal plants, exhibits multiple biological activities including anti-inflammatory effects, promotion of apoptosis, and regulation of mitochondrial metabolism. *In vitro* studies have shown that Cantleyoside acts on LPS-stimulated HFLS-RA at concentrations of 10, 20, and 40 μM, with 40 μM being the optimal dose. It inhibits cell proliferation and migration in a dose-dependent manner, reduces the production of pro-inflammatory mediators such as NO, TNF-α, IL-1β, IL-6, and MCP-1, as well as MMP-1, MMP-3, MMP-9. Additionally, it promotes apoptosis by increasing the Bax/Bcl-2 ratio. Cantleyoside induces mitochondrial dysfunction characterized by a decrease in mitochondrial membrane potential, increased levels of intracellular ROS and Ca^2+^, opening of the mitochondrial permeability transition pore, diminished activities of Na^+^-K^+^-ATPase and Ca^2+^-Mg^2+^-ATPase, and reduced oxygen consumption rate and extracellular acidification rate, leading to inhibited ATP production. Mechanistically, Cantleyoside activates the AMPK/SIRT1/NF-κB signaling pathway by upregulating AMPK phosphorylation and SIRT1 expression, inhibiting IκBα degradation, and reducing NF-κB p65 phosphorylation and nuclear translocation. This pathway activation results in suppressed inflammatory responses and enhanced apoptosis. These studies confirm Cantleyoside exerts anti-RA effects by inducing mitochondrial dysfunction, thereby activating the AMPK/SIRT1/NF-κB pathway to inhibit HFLS-RA proliferation and inflammation while promoting apoptosis (Bai et al., 2022).

Baicalin (Figure 6F), a predominant flavonoid extracted from the dry root of *Scutellaria baicalensis* Georgi and other medicinal plants, exhibits multiple biological activities, including anti-inflammatory, antioxidant, and immune-regulatory properties. Studies demonstrate that baicalin provides therapeutic benefits *in vivo* for CIA in Wistar rats. Administered intraperitoneally at doses of 50, 100, and 200 mg/kg daily for 30 days, starting from day 16 after the primary immunization, baicalin is most effective at 200 mg/kg. This dose dependently reduces the arthritis index and hind paw thickness, alleviating joint inflammation, soft tissue swelling, bone erosion, and cartilage destruction. Moreover, baicalin inhibits the secretion of pro-inflammatory cytokines TNF-α and IL-1β in rat serum and synovial tissue, showing efficacy comparable to methotrexate in this model (Methotrexate, 1 mg/kg every 3 days) at 100 mg/kg and exceeding it at 200 mg/kg. *In vitro*, baicalin impacts HFLS-RA at concentrations of 10, 20, and 30 μM, suppressing cell proliferation and reducing the secretion of TNF-α and IL-1β in culture supernatants, while modulating the NF-κB signaling pathway. Mechanistically, baicalin downregulates the expression, phosphorylation (Ser536), and acetylation (Lys310) of NF-κB p65 in both rat synovial tissue and HFLS-RA cells, while upregulating the expression of the deacetylase SIRT1, which mediates the deacetylation of NF-κB p65. These studies confirm that baicalin exerts anti-RA effects by inhibiting the NF-κB pathway through SIRT1-mediated NF-κB p65 deacetylation, thereby reducing inflammatory cytokine secretion and joint damage (Wang et al., 2014).

Ligustrazine (Figure 6G), a bioactive metabolite from *Ligusticum chuanxiong* Hort. and other medicinal plants, exhibits anti-inflammatory, antioxidant, and chondroprotective activities. It has been shown to protect against FCA-induced arthritis in rats by reducing hind-paw swelling, alleviating histopathological damages such as cartilage lesions and synovial tissue vasodilation, and improving arthritis severity. Ligustrazine dose-dependently decreases serum levels of pro-inflammatory cytokines like IL-1β, IL-6, and TNF-α, while enhancing SOD activity and reducing MDA concentration to mitigate oxidative stress. Mechanistically, it regulates the SIRT1/NF-κB pathway by reversing the FCA-induced decrease in SIRT1 expression and inhibiting NF-κBp65 and IκBα phosphorylation. It also enhances antioxidant capacity by activating the Nrf-2/HO-1 pathway, promoting Nrf-2 nuclear translocation, and increasing HO-1 expression. Previous studies found that ligustrazine protects chondrocytes from IL-1β-induced injury via the SOX9/NF-κB pathway and offers cardioprotection through the PGC-1α/Nrf2/HO-1 pathway. These findings suggest that ligustrazine can exert anti-RA effects by inhibiting inflammation, reducing oxidative stress, and protecting joint tissues through coordinated regulation of the SIRT1/NF-κB and Nrf-2/HO-1 pathways, making it a potential therapeutic candidate for arthritis (Li Y. Z. et al., 2018).

Isoliquiritigenin (Figure 6H), a flavonoid metabolite derived from *Glycyrrhiza uralensis* Fisch and other medicinal plants, exhibits a range of biological activities, including antioxidant, anti-inflammatory, and anti-apoptotic effects. Studies indicate that it provides protective effects on IL-1β (20 ng/mL) and TNF-α (20 ng/mL)-stimulated C20A4 human chondrocytes *in vitro*, at concentrations of 3, 5, and 10 μM, with 10 μM identified as the optimal dose. Isoliquiritigenin enhances cell viability in a dose-dependent manner, suppresses intracellular superoxide accumulation, and inhibits chondrocyte apoptosis by regulating apoptosis-related proteins through increased Bcl-2 expression and decreased Bax and cleaved caspase-3 levels. It also downregulates the expression of MMP13 and iNOS, while upregulating antioxidant and mitochondrial biogenesis-related proteins, including Nrf2, HO-1, SIRT1, and PGC-1α. *In vivo*, isoliquiritigenin treats AIA in Sprague-Dawley rats via intra-articular administration at a dose of 10 mg/kg for three consecutive days. This treatment improves behavior scores, reduces paw thickness and arthritis index, alleviates knee joint cartilage damage, and inhibits oxidative stress in skeletal muscles by lowering malondialdehyde and nitrotyrosine levels, as well as decreasing serum MMP13 concentration. Mechanistically, its protective effects are mediated by the Nrf2/HO-1 pathway, as inhibitors such as brusatol (Nrf2 inhibitor) and zinc protoporphyrin (Znpp, an HO-1 inhibitor) reverse its anti-apoptotic, antioxidant, and anti-inflammatory effects. These studies confirm that isoliquiritigenin exerts its anti-RA effects by activating the Nrf2/HO-1-mediated pathway to mitigate oxidative stress, inhibit chondrocyte apoptosis and extracellular matrix degradation, and alleviate joint and muscle damage (Hung et al., 2024).

Quercetin (Figure 6I), a major active flavonoid metabolite isolated from *Taxillus chinensis* (DC.) Danser and other medicinal plants, exhibits a variety of biological activities, including anti-inflammatory, antioxidant, and immune-regulatory properties. Studies have demonstrated that Quercetin provides therapeutic benefits *in vivo* for CIA in male C57BL/6 mice. It is administered orally at doses of 50 and 100 mg/kg/day, starting from day 22 post-immunization and continuing for five consecutive weeks, with the 100 mg/kg/day dose being optimal. This dosage significantly reduces clinical arthritis scores and left ankle thickness, alleviates synovial hyperplasia, inflammatory cell infiltration, pannus formation, and bone/cartilage destruction, and decreases the secretion of pro-inflammatory cytokines (IL-1β, IL-6, TNF-α, IL-8, IL-13, IL-17) in both serum and ankle joints. Mechanistically, Quercetin activates the SIRT1/PGC-1α/NRF1/TFAM signaling pathway to enhance impaired mitochondrial biogenesis and correct mitochondrial dysfunction by increasing mitochondrial DNA content and antioxidant enzyme levels (SOD, GSH-PX), while reducing malondialdehyde levels. It also inhibits the HMGB1/TLR4/p38/ERK1/2/NF-κB p65 pathway to suppress inflammatory responses by downregulating HMGB1 and TLR4 expression and the phosphorylation of p38, ERK1/2, and NF-κB p65.Clinically, epidemiological data from 30 patients with RA reveal negative correlations between plasma levels of SIRT1, PGC-1α, NRF1, and the DAS28, along with a positive correlation between HMGB1 and DAS28. These findings are consistent with its *in vivo* regulatory effects. These studies confirm that Quercetin exerts anti-RA effects by activating SIRT1 to target mitochondrial biogenesis and inhibit inflammatory signaling pathways, thereby alleviating joint pathology and restoring immune homeostasis. Quercetin has potential as a dietary therapeutic drug for RA (Shen et al., 2021).

α-Mangostin (Figure 6J), a natural isoprenyl-substituted xanthone isolated from *Garcinia mangostana* L. and other medicinal plants, exhibits various biological activities, including anti-inflammatory and immune-regulatory properties. Studies demonstrate that α-Mangostin has therapeutic effects *in vitro* on LPS-stimulated RAW264.7 cells at a concentration of 5 μg/mL. It reverses the downregulation of the α7 nicotinic cholinergic receptor and SIRT1 induced by LPS or the α7nAChR-specific inhibitor, methyllycaconitine (5 nM), thereby alleviating inflammatory responses. *In vivo*, α-Mangostin treats early-stage AIA in SD rats via intragastric administration at an optimal dose of 40 mg/kg/day. It shows better efficacy when administered early (0–20 days) compared to later stages (21–40 days). This treatment significantly inhibits M1 macrophage polarization, reducing the proportion of CD11b+CD86^+^ cells from 22.20% to 9.51%, and decreases Th1 cell differentiation, lowering the ratio of CD4+IFN-γ+ cells from 29.20% to 8.44%. Additionally, it ameliorates synovial hyperplasia, inflammatory cell infiltration, pannus formation, and articular cartilage erosion in joint tissues, while improving spleen pathological changes. Mechanistically, α-Mangostin inhibits serum acetylcholinesterase activity, increases NAD levels, and activates the cholinergic anti-inflammatory pathway by upregulating α7nAChR expression. This promotes SIRT1 activation, enhancing the deacetylation of NF-κB p65, inhibiting p65 phosphorylation, and blocking NF-κB-mediated inflammatory signaling, thereby improving the pathological immune environment in early-stage AIA rats. These studies confirm that α-Mangostin exerts anti-RA effects by regulating the CAP-SIRT1-NF-κB pathway, inhibiting M1 macrophage polarization and Th1 cell differentiation, and restoring immune homeostasis, with significant therapeutic effects particularly in the early stage of the disease (Chen W. et al., 2022).

### 5.2 TCM extracts

The ethanolic extract from the bark of *Litsea salicifolia* Roxb., a medicinal plant found in tropical and subtropical regions, contains multiple bioactive metabolites, including mesembrine, gallic acid, and quercetin-3-O-glucoside, all of which exhibit anti-inflammatory and antioxidant activities. Studies have shown that this extract provides therapeutic effects *in vitro* on LPS-stimulated RAW 264.7 murine macrophages at concentrations of 5 μg/mL and 10 μg/mL, with the latter being optimal. It dose-dependently reduces LPS-induced reactive oxygen species production, inhibits levels of nitric oxide, prostaglandin E2, and pro-inflammatory cytokines (IL-1β, IL-6, TNF-α). Additionally, it downregulates the expression of proteins such as TLR4, NF-κB p65, COX-2, and iNOS, while upregulating endogenous antioxidant molecules like Nrf2, SIRT1, and HO-1.*In vivo*, the extract attenuates Freund’s complete adjuvant-induced arthritis in male Wistar rats through oral administration at doses of 100 mg/kg and 200 mg/kg from day 1 to day 28, with 200 mg/kg being optimal. It restores body weight, reduces paw swelling and arthritis index, alleviates bone erosion and pannus formation, regulates spleen and thymus indices, and reduces oxidative and nitrosative stress by lowering MDA and NO levels, while increasing GSH content. The extract also modulates cytokine balance by decreasing levels of IL-1β, IL-6, TNF-α, IFN-γ, and increasing IL-10, alongside downregulating mRNA and protein expression of inflammatory mediators like IL-6, TGF-β, and MMP-2.Mechanistically, the extract exerts anti-RA effects by inhibiting TLR4-mediated NF-κB nuclear translocation, thereby suppressing inflammatory signaling cascades, and activating the SIRT1-Nrf2/HO-1 signaling axis to enhance antioxidant capacity. These studies confirm that the extract delivers anti-RA effects through synergistic anti-inflammatory and antioxidant actions, regulating immune function and inhibiting the activation of inflammatory pathways. This positions it as a potential therapeutic agent for chronic inflammatory diseases such as RA (Puppala et al., 2023).

### 5.3 TCM formulas

Lastly, TCM formulas such as Qufeng Epimedium Decoction exhibits multiple biological activities, such as anti-inflammation, suppression of FLS pyroptosis, and inhibition of abnormal synovial proliferation. Studies indicate that the decoction exerts therapeutic effects on RA by regulating the CYLD-SIRT1-NF-κB/GSDMD signaling pathway. *In vivo*, this decoction is administered to CFA-induced RA rats via gavage at a dose of 3.2 g/kg/day starting from day 12 after modeling, continuing for 24 consecutive days. This treatment significantly improves joint symptoms, reducing the number of swollen joints by more than 60% and the degree of joint swelling by 40% by the end of treatment. It also inhibits the secretion of pro-inflammatory cytokines (TNF-α, IL-6, IL-1β, IL-18) by 35%–60% in plasma, synovial tissue, and synovial fluid, and alleviates synovial hyperplasia and inflammatory cell infiltration, with Ki67-positive proliferating cells reduced by 55%.*In vitro*, Qufeng Epimedium Decoction inhibits pyroptosis of FLS isolated from RA model rats, decreasing the proportion of Caspase-1 and Propidium Iodide double positive pyroptotic cells from 10.22% to 4.21%. Mechanistically, it upregulates the expression of the deubiquitinase CYLD, which interacts with SIRT1 to mediate its deubiquitination and protein stabilization, reducing SIRT1 ubiquitination levels by 45%. This action deactivates the NF-κB/GSDMD signaling pathway, with phosphorylated NF-κB p65 expression reduced by 30%, and downregulates mRNA and protein levels of pyroptosis-related molecules (Caspase-1, GSDMD, NLRP3) by 50%–70%. These findings confirm that Qufeng Epimedium Decoction exerts anti-RA effects by inhibiting inflammation, suppressing FLS pyroptosis, and regulating the CYLD-SIRT1-NF-κB/GSDMD signaling pathway. This provides an experimental basis for its clinical application in RA treatment (Wu et al., 2025).

Bushen Jiedu Tongluo Formula possesses multiple biological activities, including anti-inflammatory and anti-angiogenic effects.Studies have shown that this formula exerts therapeutic effects on CIA in female SD rats through intragastric administration at concentrations of 0.98 g/mL, 1.95 g/mL, and 3.9 g/mL for 21 consecutive days, with 3.9 g/mL identified as the optimal dose. Its efficacy is comparable to that of the SIRT1 agonist SRT1720 (0.1 g/kg/day), used as a positive control. The formula significantly improves arthritis symptoms, reduces toe thickness and arthritis index, inhibits synovial hyperplasia and inflammatory cell infiltration, prevents pannus formation, decreases synovial microvessel density, and CD31^+^ expression, and downregulates levels of pro-inflammatory factors while reducing acetylation levels of HMGB1 and HIF-1α in synovial tissue. *In vitro*, Bushen Jiedu Tongluo Formula inhibits the proliferation, migration, and capillary-like tube formation of HMGB1 (100 ng/mL)-induced HUVECs at safe concentrations of 0.25, 0.5, and 1 mg/mL. Mechanistically, it upregulates SIRT1 expression at both protein and mRNA levels, while downregulating the expression of HMGB1, NF-κB p65, HIF-1α, and VEGF, thereby blocking the cascade of signaling pathway. These studies demonstrate that Bushen Jiedu Tongluo Formula exerts anti-RA effects by inhibiting synovial inflammation, suppressing angiogenesis, and regulating the SIRT1-mediated HMGB1/NF-κB/HIF-1α/VEGF pathway, with dose-dependent efficacy (Chen, 2021).

Despite their different focuses, Qufeng Epimedium Decoction and Bushen Jiedu Tongluo Formula both use SIRT1 as a key regulatory hub. Qufeng Epimedium Decoction enhances SIRT1 stability via CYLD, while Bushen Jiedu Tongluo Formula upregulates SIRT1 expression. Both actions reduce NF-κB signaling, leading to lower levels of pro-inflammatory cytokines like TNF-α, IL-6, and IL-1β, which helps alleviate synovial inflammation (Figure 3). Specifically, Qufeng Epimedium Decoction inhibits FLS pyroptosis through the NF-κB/GSDMD pathway, whereas Bushen Jiedu Tongluo Formula curbs angiogenesis via the SIRT1/HMGB1/HIF-1α/VEGF axis. Together, they address crucial RA pathologies, such as synovial hyperplasia and tissue destruction. Their multi-component nature enables modulation of SIRT1, NF-κB, and key disease processes, thus overcoming the limitations of single-target therapies in RA. This synergistic regulation reflects TCM’s holistic philosophy and highlights its benefits in RA management and the potential development of multi-target therapeutics.

### 5.4 Translational readiness

TCM-derived HDAC activators, particularly those targeting the class III HDAC isoform SIRT1, such as resveratrol, Bushen Jiedu Tongluo Formula, ligustrazine, Qufeng Epimedium Decoction, *L. salicifolia* Roxb. extract, quercetin, baicalin, α-mangostin, Wutou Decoction, cantleyoside, and isoliquiritigenin, show significant potential for RA therapy backed by robust preclinical evidence. These agents have been validated *in vitro* using RA-relevant models to activate SIRT1, thereby suppressing pro-inflammatory signaling pathways, inhibiting FLS proliferation and pyroptosis, synovial angiogenesis, and osteoclastogenesis, and reducing oxidative stress. Mechanistically, they exert distinct SIRT1-dependent effects, such as Qufeng Epimedium Decoction stabilizing SIRT1 through CYLD-mediated deubiquitination and Wutou Decoction mediating HMGB1/NF-κB deacetylation. *In vivo* studies have shown efficacy in CIA and AIA models, improving arthritis symptoms and aligning mechanistically with *in vitro* findings. Resveratrol and baicalin have advanced to human-derived validations, demonstrating SIRT1-mediated anti-inflammatory effects. However, their clinical translation faces significant hurdles. The absence of structured clinical trials leaves their safety and pharmacokinetic profiles in humans unconfirmed. Despite their multi-target synergy and favorable safety profiles, these agents face translational challenges due to the lack of clinical trials, limited HDAC isoform specificity, standardization issues for TCM decoctions, and incomplete bioavailability data for monomeric metabolites. To accelerate translation, future research focus on conducting early-phase clinical trials with precise biomarker endpoints, enhancing formulation specificity and bioavailability, standardizing decoctions, and broadening *ex vivo* human validations. Addressing these challenges will facilitate the progress of TCM-derived SIRT1 activators toward effective clinical application in RA treatment.

## 6 Dual modulators: balancing pro-inflammatory and anti-inflammatory HDACs

While most metabolites selectively inhibit or activate HDAC isoforms, Wutou Decoction functions as a dual HDAC modulator, exerting both inhibitory and activating actions. Wutou Decoction contains a variety of chemical metabolites including alkaloids, flavonoids, saponins, and monoterpenoid glycosides. Notable metabolites are benzoylaconitine, benzoylmesaconine, benzoylhypaconitine, aconitine, ephedrine, and pseudoephedrine hydrochloride from the alkaloid category; liquiritin from flavonoids; astragaloside IV and glycyrrhizic acid from saponins; and paeoniflorin from monoterpenoid glycosides. In the context of HDAC and SIRT1 studies, network pharmacology predictions and molecular docking analyses suggest that ephedrine might interact with HDAC7 through low-binding-energy interactions, indicating its potential role in regulating HDAC7 expression. Paeoniflorin and aconitine derivatives are presumed to have anti-inflammatory properties and selectively inhibit pro-inflammatory HDAC subtypes. These metabolites may synergistically activate SIRT1, thereby providing anti-RA effects through deacetylation modifications of HMGB1 and NF-κB p65. Related effects have been verified, supporting their potential therapeutic role in RA treatment (Ba, 2020; Shen et al., 2025).

Wutou Decoction exerts anti-RA effects through a dual regulatory mode, inhibiting pro-inflammatory HDAC1/7 while activating anti-inflammatory SIRT1. By inhibiting HDAC1/7, it blocks pathological proliferation, cartilage damage, and abnormal activation of synovial cells. Studies have demonstrated that Wutou Decoction reduces HDAC7 mRNA levels by 45% in TNF-α-induced RA FLS, suppressing abnormal cell proliferation, migration, and angiogenesis, which helps prevent synovial pannus formation. In CIA rat models, it significantly downregulates HDAC7 expression in synovial tissue, reducing synovial hyperplasia, inflammatory cell infiltration, and alleviating joint swelling and inflammatory responses (Ba, 2020). Simultaneously, Wutou Decoction activates SIRT1, enhancing anti-inflammatory responses. It doubles SIRT1 deacetylase activity in LPS-induced RAW264.7 macrophages, promoting deacetylation of HMGB1 and NF-κB p65, thus suppressing their pro-inflammatory functions. In CIA rat models, it upregulates SIRT1 expression in synovial tissue, inhibiting the infiltration and activation of M1 macrophages (marked by F4/80+iNOS+), thereby mitigating articular bone and cartilage damage, and this effect can be reversed by the SIRT1 inhibitor EX527 (Shen et al., 2025). Moreover, Wutou Decoction induces the expression of the long non-coding RNA LOC101928120, which binds to HDAC1 through its 100–400 bp region. This interaction decreases histone H3 acetylation levels in the promoter region of the SHC1 gene, inhibiting SHC1 transcription and expression. As a result, reactive oxygen species production and matrix metalloproteinase-13 secretion in chondrocytes are reduced, alleviating cartilage degradation (Wu D. et al., 2021). This comprehensive mechanism underscores the therapeutic potential of Wutou Decoction in RA treatment through targeted modulation of HDAC and SIRT1 pathways.

Despite the preliminary validation of Wutou Decoction dual regulatory mechanism, several limitations persist in current research. Studies mainly focus on changes in HDAC1/7 mRNA and protein levels without directly measuring their enzymatic activity using fluorometric substrate assays. This leaves uncertainty about whether Wutou Decoction directly inhibits enzyme activity or indirectly regulates gene transcription. Comparative experiments between single-target blockade (such as HDAC7 siRNA silencing or SIRT1 inhibition by EX527) and dual-target blockade have not been conducted, preventing quantification of the respective contributions and synergistic effects of HDAC1/7 inhibition and SIRT1 activation. To enhance the scientific rigor and translational potential of Wutou Decoction research, future studies should employ HDAC activity assay kits to measure the direct inhibitory effects of Wutou Decoction and its metabolites on HDAC1/7. Immunoprecipitation techniques should be used to validate direct binding and deacetylation between SIRT1 and HMGB1/NF-κB p65. Additionally, single-target and dual-target blocking experiments, quantified by the Bliss method, are necessary to understand the contribution of each mechanism. Isolating and purifying individual metabolites will help test their specific effects on HDAC1/7 and SIRT1. Batch consistency studies of Wutou Decoction extracts should clarify the relationship between metabolite content and efficacy, while toxicity assessments on normal synovial cells, chondrocytes, and major organs will define the safety window and optimize administration. Further studies should use primary FLS and synovial tissue samples from RA patients to validate Wutou Decoction’s effects in human cells. Measuring HDAC subtypes and SIRT1 expression in RA patient serum will help analyze correlations with disease phenotypes, improving clinical translation. Examining Wutou Decoction’s combined effects with clinical drugs like Methotrexate will evaluate synergistic potential and safety, preparing for future clinical trials.

## 7 Quality control, exposure and safety considerations for TCM-derived HDAC modulators

HDAC modulators derived from TCM offer significant therapeutic potential for RA by targeting multiple inflammatory pathways, regulating oxidative stress, and maintaining joint integrity. However, translating these natural products into clinical use faces challenges such as inadequate standardization, low *in vivo* exposure, and insufficient safety evidence. A key concern is the frequent presence of PAINS, which can cause false positives in bioassays by non-specifically interfering through mechanisms like metal chelation or redox activity. Many TCM metabolites contain PAINS-defining substructures, and most PAINS meet druglikeness criteria, making them hard to exclude with conventional filters. To address this challenge and rigorously distinguish genuine HDAC modulators, we recommend a systematic three-step validation strategy. First, computational PAINS filtering should be performed, supplemented by Tanimoto Interaction Similarity analysis to assess PAINS-associated binding mode conservation. Second, orthogonal bioassays with appropriate interference controls should be conducted to rule out non-specific effects. Third, mechanistic validation using HDAC isoform-specific gene-silencing approaches is essential (Bolz et al., 2021). This systematic approach helps distinguish true HDAC modulators from PAINS, facilitating the development of safe, effective TCM-based therapies for RA.

Quality control is a major difficulty due to the complexity of TCM metabolites, which results in batch-to-batch variability affecting therapeutic consistency. To mitigate this, advanced analytical techniques should be employed. These techniques can establish chemical fingerprint profiles for raw materials and final products, allowing for qualitative identification and quantitative traceability of active metabolites. To ensure biological activity consistency, it is essential to establish enzyme-level *in vitro* HDAC inhibition/activation assays and cellular-level epigenetic regulation validation based on HDAC mechanisms. Comprehensive SOPs and traceability management throughout the production chain can reduce source-level variability (Pan et al., 2020). Quantitative metrics, such as fingerprint similarity between batches, active metabolite content fluctuation ranges, and bioactivity reproducibility, should be used to achieve precise quality control (Liu C. et al., 2025). These measures will help ensure the reliability and effectiveness of HDAC modulators in RA treatment and support their transition into standardized therapeutic agents.

Optimizing *in vivo* exposure profiles of HDAC modulators is constrained by low oral bioavailability, largely due to poor water solubility, limited intestinal mucosal permeability, and degradation from gut microbiota metabolism and hepatic first-pass effects. Addressing these issues requires a blend of modern pharmaceutical technologies with PK strategies. To enhance solubility, *in vivo* stability, and transport efficiency across intestinal epithelial cells, innovative techniques such as nanodelivery systems, prodrug modification, and phospholipid complex preparations can be employed (Dwivedi et al., 2024). These methods aim to improve the pharmacokinetic profile and therapeutic efficiency of active metabolites. Comprehensive PK studies should be conducted across preclinical animal models and extend into clinical trials to thoroughly understand the absorption, distribution, metabolism, and excretion characteristics of these metabolites and their metabolites. These studies are crucial to elucidate the dose-response relationship and to determine whether the therapeutic activity is primarily due to the parent metabolite or its metabolites. This information will provide a scientific basis for personalized optimization of clinical dosing regimens, enhancing the therapeutic potential of HDAC modulators in rheumatoid arthritis treatment.

Integrating long-standing clinical experience with TCM and modern evidence-based validation is essential for the safety assessment of HDAC modulators. Key concerns include off-target effects as epigenetic regulators, DDI risks with conventional RA therapies, and a lack of internationally standardized clinical safety data. Addressing these requires a comprehensive approach across preclinical and clinical phases: conduct preclinical toxicology studies to evaluate non-target tissue HDAC activity disruption, genome-wide expression changes, and long-term toxicity. Investigate DDIs to clarify pharmacokinetic and pharmacodynamic interactions with anti-rheumatic drugs and define safe thresholds for synergy. Design randomized controlled trials with accurate control groups and objective efficacy endpoints such as DAS28 scores, joint imaging, and cytokine levels, while simultaneously monitoring hepatic and renal function, hematological parameters, immune function, and adverse events. This will allow scientific evaluation of the clinical benefit-risk ratio. In summary, translating TCM-derived HDAC modulators into clinical use requires establishing standardized quality control, optimizing delivery technologies, and conducting rigorous safety validations. This multidisciplinary integration aims to transition natural products into reliable therapeutics and offer innovative strategies for personalized RA treatment.

## 8 Conclusion

HDACs exert isoform-specific roles in RA pathogenesis: pro-inflammatory subtypes (e.g., HDAC1, 2, 3, 6) drive FLS hyperplasia, amplify pro-inflammatory cytokine secretion, and promote osteoclast-mediated bone erosion, while anti-inflammatory isoforms (e.g., HDAC4, 5, SIRT1) support immune tolerance restoration and bone homeostasis maintenance. This functional duality highlights the necessity of moving beyond non-selective pan-HDAC inhibition toward precise, isoform-targeted modulation to balance therapeutic efficacy and minimal off-target toxicity. TCM emerges as a promising source of naturally derived HDAC modulators, with its bioactive constituents characterized by structural diversity, moderate potency, and low toxicity—key advantages for long-term RA management. Notably, certain TCM-derived metabolites can selectively inhibit pro-inflammatory HDACs while activating protective subtypes, rebalancing immune responses and alleviating chronic synovial inflammation in RA. At the molecular level, TCM-mediated HDAC modulation converges on key deacetylation substrates, including transcription factors (NF-κB p65, AP-1, STAT3, NLRP3, HMGB1, HIF-1α) and histone H3, with collective effects in attenuating RA-related inflammation, synovial angiogenesis, and metabolic dysregulation.

Despite substantial preclinical advancements, critical challenges persist in the research and translation of TCM-derived HDAC modulators. Future research should prioritize five focused directions. First, optimize clinically relevant models (e.g., patient-derived organoids, 3D synovial cultures) and integrate multi-omics (epigenomics, metabolomics) with genetic manipulation tools to validate causal signaling pathways and enhance mechanistic rigor. Second, implement a systematic PAINS validation workflow by deploying computational tools to identify PAINS-defining substructures, verifying target specificity through orthogonal bioassays integrated with interference controls and ChIP-based validation of HDAC-substrate binding to exclude false positives. Third, conduct comprehensive PK profiling and long-term safety evaluations to define therapeutic windows for TCM metabolites and formulations. Fourth, standardize TCM formulas via HPLC-MS-based quantitative analysis of bioactive constituents, with a focus on multi-component synergistic interactions. Fifth, design biomarker-driven phase I/II clinical trials stratified by disease activity, serological subtype, and prior medication history, while exploring synergistic effects with conventional DMARDs or biologic therapies. In conclusion, TCM-derived HDAC modulators serve as multi-target epigenetic regulators, integrating traditional herbal medicine with modern epigenetic drug development paradigms and offering a promising avenue for RA treatment.

## Glossary

ACPA: Anti-Citrullinated Protein Antibodies
ADAMTS-5: A Disintegrin and Metalloproteinase with Thrombospondin Motifs-5
AIA: Adjuvant-Induced Arthritis
Akt: Protein Kinase B
AMPK: AMP-Activated Protein Kinase
AP-1: Activator Protein-1
ATP: Adenosine Triphosphate
Bax: Bcl-2-Associated X Protein
Bcl-2: B-Cell Lymphoma-2
Blimp-1: B Lymphocyte-Induced Maturation Protein 1
caspase-1: Cysteine-Aspartic Acid Protease 1
CIA: Collagen-Induced Arthritis
C20A4: Human Chondrocyte Cell Line C20A4
CCL: C-C Motif Chemokine Ligand
CCR: C-C Motif Chemokine Receptor
ChIP: Chromatin Immunoprecipitation
CIRP: Cold-Inducible RNA-Binding Protein
CMA: chaperone-mediated autophagy
COL2A1: Collagen Type II Alpha 1 Chain
COX-2: Cyclooxygenase-2
Cul3: Cullin 3
CYLD: Cylindromatosis Tumor Suppressor
CXCL: C-X-C Motif Chemokine Ligand
DAS28: Disease Activity Score in 28 Joints
ERK1/2: Extracellular-Signal-Regulated Kinase 1/2
FLS: Fibroblast-Like Synoviocyte
FOXK1: Forkhead Box K1
FOXP3: Forkhead Box P3
GSDMD: Gasdermin D
GSH-Px: Glutathione Peroxidase
HAT: Histone Acetyltransferase
HDAC: Histone Deacetylase
HDACi: Histone Deacetylase inhibitor
HFLS-RA: Human Fibroblast-Like Synoviocyte from Rheumatoid Arthritis
HIF-1α: Hypoxia-Inducible Factor-1α
HMGB1: High Mobility Group Box 1
HO-1: Heme Oxygenase-1
Hsc70: Heat shock cognate protein 70
HSP90AA1: Heat Shock Protein 90 Alpha Family Class A Member 1
HUVEC: Human Umbilical Vein Endothelial Cell
IκBα: Inhibitor of NF-κB Alpha
IKBκB: Inhibitor of Nuclear Factor Kappa B Kinase Subunit Beta
IKK: IκB Kinase
IFN: Interferon
IL-10: Interleukin-10
IL-1β: Interleukin-1 Beta
IL-6: Interleukin-6
IL-8: Interleukin-8
IRF1: Interferon Regulatory Factor 1
iNOS: Inducible Nitric Oxide Synthase
Keap1: Kelch-Like ECH-Associated Protein 1
LAMP2A: lysosome-associated membrane protein type 2A
lncRNA: Long-Stranded Non-Coding RNA
MCP-1: Monocyte Chemoattractant Protein-1
MAPK: Mitogen-Activated Protein Kinases
MDA: Malondialdehyde
M-CSF: Macrophage Colony-Stimulating Factor
MH7A: Human Fibroblast-Like Synoviocyte Cell Line
MTA1: Metastasis-Associated Protein 1
MyD88: Myeloid Differentiation Primary Response 88
**NAD** ^**+**^: Nicotinamide-Adenine-Dinucleotide
NFKB2: Nuclear Factor Kappa B Subunit 2CFA
NF-κB p65: Nuclear factor kappa-light-chain-enhancer of activated B cells p65
Nrf2: Nuclear Factor-Erythroid 2-Related Factor 2
NLRP3: NOD-Like Receptor Protein 3
NO: Nitric Oxide
Nur77: Nuclear Receptor Subfamily 4 Group A Member 1
OA: Osteoarthritis
OPG: Osteoprotegerin
P-p65: phosphorylated p65
Pax5: Paired Box 5
PAINS: Pan-assay interference compounds
PBMCs: Peripheral Blood Mononuclear Cells
PGC-1α: Peroxisome Proliferator-Activated Receptor Gamma Coactivator 1α
PI3K: Phosphoinositide 3-Kinase
PK/PD: Pharmacokinetics/Pharmacodynamics
Prdm1: PR Domain Zinc Finger Protein 1
**PGE**_**2**_: Prostaglandin E_2_
p38 MAPK: p38 Mitogen-Activated Protein Kinase
RA: Rheumatoid Arthritis
RAW264.7: Mouse Macrophage Cell Line RAW264.7
RANK: Receptor Activator of Nuclear Factor Kappa-B
RANKL: Receptor Activator of Nuclear Factor Kappa-B Ligand
RORc: RA-Related Orphan Receptor Gamma
ROS: Reactive Oxygen Species
Runx2/Cbfa-1: Runt-Related Transcription Factor 2/Core Binding Factor Alpha 1
S1P: Sphingosine-1-Phosphate
siRNA: Small Interfering RNA
SHC1: SHC Adaptor Protein 1
SIR2: silencing information regulator 2
SOCS3: Suppressor of Cytokine Signaling 3
smad: Sma and Mad related family
SOD: Superoxide Dismutase
SphK1: Sphingosine Kinase 1
S1PR1: Sphingosine-1-Phosphate Receptor 1
SD: Sprague-Dawley Rat Strain
SCFA: Short-Chain Fatty Acid
STAT3: Signal Transducer and Activator of Transcription 3
TGF-β1: Transforming Growth Factor-β1
TFAM: Mitochondrial Transcription Factor A
THP-1: Human Monocytic Cell Line THP-1
TNF-α: Tumor Necrosis Factor-α
TRAF6: TNF Receptor-Associated Factor 6
TRAP: Tartrate-Resistant Acid Phosphatase
TLR4: Toll-Like Receptor 4
TCM: Traditional Chinese Medicine
Th1: T Helper 1 Cell
Th2: T Helper 2 Cell
Th17: T Helper 17 Cell
Treg: Regulatory T Cell
VEGF: Vascular Endothelial Growth Factor
VEGFR2: Vascular Endothelial Growth Factor Receptor 2
ZO-1: Zonula Occludens-1.
